# Associations of dietary, lifestyle, and other participant characteristics with APC, β-catenin, E-cadherin, and MSH2 expression in the normal mucosa of sporadic colorectal adenoma patients

**DOI:** 10.3389/fgstr.2022.889925

**Published:** 2022-11-07

**Authors:** Timothy D. Shu, Robin E. Rutherford, March E. Seabrook, Elizabeth L. Barry, Roberd M. Bostick

**Affiliations:** ^1^ Department of Epidemiology, Rollins School of Public Health, Emory University, Atlanta, GA, United States; ^2^ Division of Digestive Diseases, Department of Medicine, School of Medicine, Emory University, Atlanta, GA, United States; ^3^ Consultants in Gastroenterology, West Columbia, SC, United States; ^4^ Department of Epidemiology, Geisel School of Medicine at Dartmouth, Lebanon, NH, United States; ^5^ Winship Cancer Institute, Emory University, Atlanta, GA, United States

**Keywords:** APC, beta-catenin, E-cadherin, MutS homolog 2, population characteristics, colorectal neoplasms, cross-sectional studies

## Abstract

Abnormal expression of Wnt pathway and DNA mismatch repair proteins is common during colorectal carcinogenesis. To investigate cross-sectional associations of lifestyle, dietary, and other participant characteristics with the expression of such proteins, we measured APC, β-catenin, E-cadherin, and MSH2 colorectal crypt expression in biopsies of normal-appearing colorectal mucosa from 104 sporadic colorectal adenoma patients using automated immunohistochemistry and quantitative image analysis. We used multivariable general linear models to compare adjusted mean biomarker expression across categories of participant characteristics. Example findings include that among women relative to men, mean APC expression in whole crypts, the upper 40% of crypts (differentiation zone), and the lower 60% of crypts (proliferation zone) was 322.9% higher (p<0.01), 296.7% higher (p<0.01), and 399.1% higher (p<0.01), respectively. Among participants with higher alcohol consumption, APC expression in the crypt differentiation zone was estimated to be 15.9% lower (p=0.08). Among those with higher total meat consumption, β-catenin expression in whole crypts and the crypt proliferation zone was estimated to be 20.5% higher (p=0.07) and 19.6% higher (p=0.06), respectively, and MSH2 expression in the crypt differentiation zone was estimated to be 64.4% lower (p=0.10). Among those with a higher body mass index, MSH2 expression in the crypt differentiation zone was estimated to be 87.5% lower (p=0.15). These pilot study findings suggest that being male, higher adiposity, and higher alcohol and meat consumption may be unfavorably associated with biomarkers of colorectal carcinogenesis pathway proteins in the normal-appearing colorectal mucosa of sporadic colorectal adenoma patients and support further investigation in larger studies.

## Introduction

Colorectal cancer (CRC) is the second leading cause of cancer-related death in the US among men and women combined (1, 2). Considerable evidence supports that a large proportion of CRC incidence may be related to a combination of environmental exposures, such as diet, smoking, alcohol consumption, and a sedentary lifestyle (3). Considerable evidence also supports that most colorectal cancers develop through changes (mutations, DNA methylation, expression levels) in certain Wnt signaling pathway (in about 85% of sporadic colorectal cancers) and DNA mismatch repair (MMR) genes (in about 15% of sporadic colorectal cancers) (4, 5). These genes are involved in the normal structure and function of colorectal crypts, and become altered during colorectal carcinogenesis (6). The protein products of these genes include APC, β-catenin, and E-cadherin in the Wnt pathway, and the MSH2 and MLH1 MMR proteins (6, 7). The expression levels of these proteins in whole crypts and in functional crypt zones, such as the proliferation zone (lower 60% of crypts) and the differentiation zone (upper 40% of crypts) in the normal mucosa were reported to differ between patients with incident, sporadic colorectal adenoma (the immediate precursor to most CRCs), and those with no current or previous adenoma (8, 9). The expression levels of these proteins have also been reported to be modifiable by calcium and/or vitamin D supplementation in randomized controlled trials (10–12). These findings support that these proteins may serve as modifiable, pre-neoplastic, biomarkers of risk for colorectal neoplasms. Other than testing the effects of calcium and vitamin D on the biomarkers, there has been limited investigation of associations of other dietary, lifestyle, and other participant characteristics with the biomarkers (8, 9). Detection of such associations would provide clues for exposures that may modify the expression of the biomarkers and reduce risk for colorectal neoplasms.

Accordingly, we investigated cross-sectional differences in expression of APC, β-catenin, E-cadherin, and MSH2 (which was highly correlated with MLH1 expression in our previous studies (9, 12)) across categories of multiple demographic, medical, dietary, and lifestyle characteristics among sporadic colorectal adenoma patients participating in a randomized, controlled trial of calcium and/or vitamin D and colorectal adenoma recurrence. We hypothesized that putative anti-colorectal cancer risk factors would be associated with higher APC, E-cadherin, and MSH2 expression, and lower total β-catenin expression, as well as higher anti-proliferative APC relative to pro-proliferative β-catenin expression, and higher MSH2 relative to mib-1 (epitope of the Ki-67 proliferation marker) expression (i.e., higher mismatch repair relative to proliferation). We also hypothesized that the directions of associations of putative pro-colorectal cancer risk factors with the expression levels of the biomarkers would be opposite to those for the anti-colorectal cancer risk factors.

## Materials and methods

### Participant population

Study participants were recruited from two clinical centers as part of a larger randomized, placebo-controlled, chemoprevention clinical trial testing the efficacy of supplemental calcium and vitamin D for preventing colorectal adenoma recurrence (the “parent” study, NCT00153816) (13). Eligible participants were 45 – 75 years old, in general good health, and within 4 months of study entry had a complete, clean colonoscopy reaching the cecum resulting in a histologically-identified neoplastic polyp ≥2 mm in diameter from any site in the colon or rectum. Exclusion criteria included an invasive carcinoma in any polyp removed, familial colonic polyposis syndromes, inflammatory bowel diseases, malabsorption syndromes, history of large bowel resection, alcohol or narcotic dependence, serum calcium concentration outside the normal range, serum creatinine >20% concentration over the upper limit of normal, serum 25-hydroxy-vitamin D (25[OH]D) concentration <12 ng/mL (30 mmol/L or 0.00012 kg/m^3^) or >90 ng/mL (225 mmol/L or 0.00090 kg/m^3^), history of kidney stones or hyperparathyroidism, and history of osteoporosis or other medical condition that could require supplemental vitamin D or calcium. Additional exclusion criteria for the adjunct biomarker study included being unable to be off aspirin for 7 days, history of a bleeding disorder, or current use of an anticoagulant medication.

### Parent study

For the parent study (13), between May 2004 and July 2008, 19,083 apparently eligible patients were identified through initial screening of colonoscopy and pathology reports. Of these, 2,259 met the final eligibility criteria, consented to participate, and were randomized. After the parent study was underway, funding was received for the adjunct biomarker study. For the adjunct biomarker study (11, 14), prior to randomization, 231 apparently eligible parent study participants at two of the 11 clinical centers (South Carolina and Georgia) were offered participation in the biomarker study. Of these, 109 met the final eligibility criteria, signed consent, and had baseline rectal biopsies taken; of these, sufficient biopsy tissue for biomarker measurements was obtained at baseline on 104. All participants signed a consent form upon enrollment. The Institutional Review Board (IRB) for both clinical centers (the Emory IRB) approved the research (Emory IRB numbers IRB00012627 for the parent study [original approval February 16, 2004 and renewed annually] and IRB00000357 for the adjunct biomarker study [original approval October 14, 2005, and renewed annually]).

At enrollment, study coordinators collected from each study participant information on medical history, medication and nutritional supplement use, diet, and lifestyle. Diet was assessed using a semi-quantitative Block Brief 2000 food frequency questionnaire (FFQ) (NutritionQuest, Berkeley, CA). Physical activity was assessed and categorized as low, moderate, and high using the short form International Physical Activity Questionnaire (IPAQ; publicly available at www.ipaq.ki.se). Peripheral venous blood samples for measurements of calcium, creatinine, and 25(OH)D concentrations were also obtained at baseline (13).

### Biopsy collection 

Adjunct biomarker study participants underwent biopsies of normal-appearing rectal mucosa without any preceding bowel-cleansing procedure (11, 14). Six 1 mm-thick biopsies were collected from the rectal mucosa 10 cm above the external anal aperture through a proctoscope using jumbo cup flexible biopsy forceps. No biopsy was taken within 4.0 cm of a polypoid lesion. Biopsies were immediately placed into saline, oriented, transferred to 10% normal-buffered formalin for 24 hours, then transferred to 70% ethanol for up to a week, and then embedded in paraffin blocks (two blocks of three biopsies per participant, per visit). APC, β-catenin, E-cadherin, and MSH2 (plus mib-1 [Ki-67 epitope]) were measured in the biopsies using automated immunohistochemistry with image analysis (mib-1 was included for the present analyses as a marker of proliferation, strictly to assess MSH2 expression relative to proliferation) (11, 14).

### Immunohistochemistry protocol

Five slides with three levels of 3 μm-thick biopsy sections taken 40 μm apart were prepared for each biomarker. Heat-mediated antigen retrieval was then used to uncover the epitope. Slides were placed into a preheated Pretreatment Module (Lab Vision Corp., Fremont, CA) with 100x citrate buffer pH 6.0 (DAKO S1699, DAKO Corp., Carpinteria, CA) and steamed for 40 minutes. The slides were then put into a DakoCytomation Autostainer Plus system that immunohistochemically processed the slides using a streptavidin-biotin method (LSAB2 Detection System [DAKO K0675]) and a monoclonal antibody to each biomarker. Commercially available antibodies were selected based on previous literature and comparing the staining patterns found in positive and negative control tissues and in normal colorectal mucosa with those reported in the literature in which antibody specificity was validated. Information on the antibodies is as follows. For APC: antibody ID – AB_2057371; antibody name – anti-APC (Ab-7) mouse mAb (CC-1) antibody; clone ID – CC-1; clonality – monoclonal; target antigen – APC human; vendor/catalog number – Millipore OP80; and host organism – mouse. For β-catenin: antibody ID – AB_397555; antibody name – mouse anti-catenin beta monoclonal antibody unconjugated clone 14; clone ID – 14; clonality – monoclonal; target antigen – human catenin; vendor/catalog number – BD Biosciences 610154; and host organism – mouse. For E-cadherin: antibody ID AB_2533118; antibody name – E-cadherin monoclonal antibody (4A2C7); clonality – monoclonal; target antigen – E-cadherin human; vendor/catalog number – Thermo Fisher 33-4000; and host organism – mouse. For MSH2: antibody ID – AB_2266524; antibody name – anti-MSH2 (Ab-2) mouse mAb (FE11) antibody; clonality – monoclonal; target antigen – MSH2 human; vendor/catalog number – Millipore NA27; and host organism – mouse. For mib-1: antibody ID – AB_2142367; antibody name – Ki-67 antibody; clone ID – mib-1; clonality – monoclonal; target antigen – human recombinant peptide corresponding to a 1002 bp Ki-67 cDNA fragment; vendor/catalog number – DAKO M7240; and host organism – mouse. The antibodies were used in the following concentrations: APC – 1:50; β-catenin – 1:300; E-cadherin – 1:50; MSH2 – 1:500; and mib-1 – 1:350. Only the slides for MSH2 were counterstained (with hematoxylin); all slides were cover-slipped with a Leica CV5000 Coverslipper (Leica Microsystems, Inc., Buffalo Grove, IL). Positive and negative control slides were included in each staining batch (11, 14).

### Quantifying labeling densities of biomarkers in normal colorectal crypts

**Figure 1:**
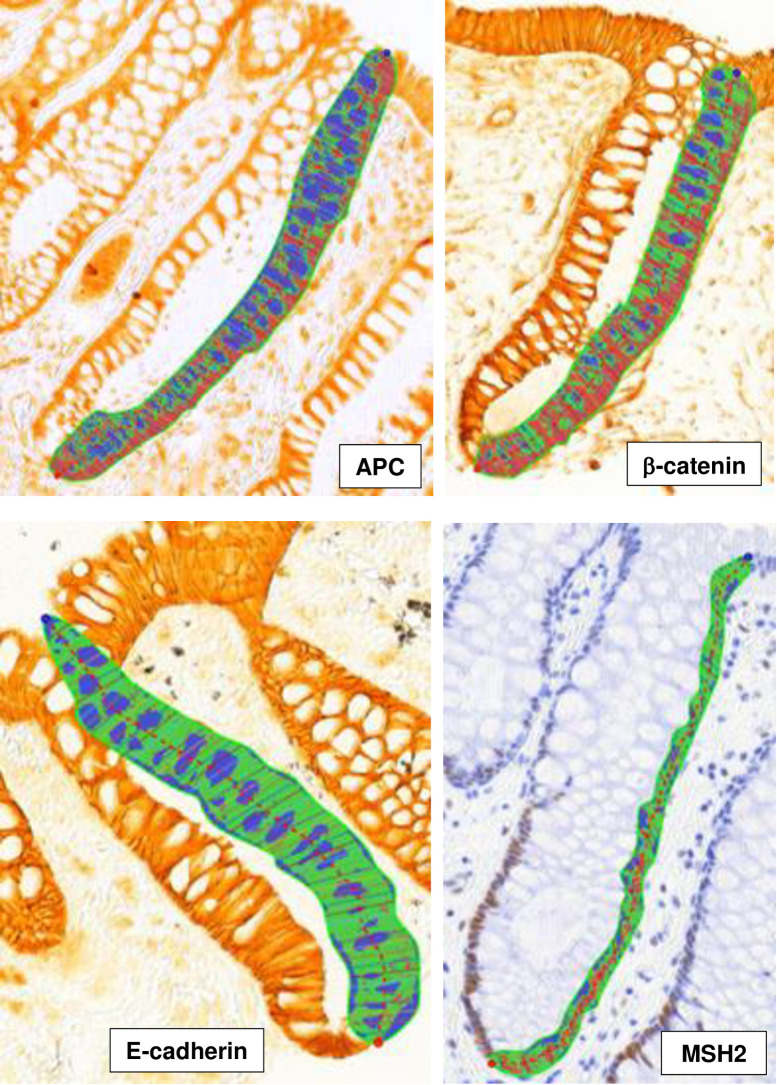
Images of colorectal crypts in histologic sections of biopsies of morphologically-normal rectal mucosa, immunohistochemically processed for APC, β-catenin, E-cadherin, and MSH2 proteins. Each image also shows a hemicrypt that was traced by the laboratory technician, segmented by the image analysis program, and the biomarker labeling identified and its labeling optical density measured in the entire hemicrypt and in its segments by the image analysis program. Note that for APC, β-catenin, E-cadherin, no counterstain was used, and the entire epithelium of the hemicrypts was traced, and for MSH2, counterstaining with hematoxylin was used, and only the nuclear zone of the hemicrypts was traced.

The optical densities of the immunohistochemically-labeled biomarkers in colorectal crypts were measured using quantitative image analysis (**Figure 1**). First, slides were scanned using an Aperio Scanscope CS digital scanner (Aperio Technologies, Inc., Vista, CA). Next, these digital images were reviewed with our custom-developed CellularEyes program (DivEyes LLC, Atlanta, GA) to identify colorectal crypts that would be acceptable for analysis. A “scorable” crypt was defined as an intact crypt that extended from the muscularis mucosa to the colorectal lumen. Before analysis, images of the negative and positive control slides were checked to ensure staining adequacy. Standardized settings were used for all equipment throughout the scoring process. The technician, who was blinded to participant characteristics, selected two of three biopsies with 16 – 20 “scorable” “hemicrypts” (one side of the crypt) per biopsy and used a digital drawing board to trace the border of each hemicrypt. The program then divided the hemicrypt outline into 50 equally spaced segments (with the average widths of normal colonocytes) and measured the background-corrected optical density of the biomarker labeling across the entire hemicrypt and for each individual segment. Our measurement technology did not allow for measuring biomarker subcellular locations. Resulting data were automatically transferred into a MySQL database (Sun Microsystems Inc., Redwood Shores, CA). These analysis steps were then repeated for the next identified hemicrypt. Using these procedures, we analyzed, on average, approximately 28 hemicrypts/biomarker/participant, yielding a total of approximately 2,900 hemicrypts analyzed per biomarker across all participants. Reliability control samples that were previously analyzed by the reader were re-analyzed over the course of the trial to determine intra-reader “scoring” reliability. The intra-class correlation coefficient for each biomarker was >0.90 (11, 14).

### Statistical analysis

All analyses were cross-sectional, conducted on baseline data only. We summarized participant characteristics using simple descriptive statistics, such as means, ranges, and standard deviations for continuous variables, and proportions as percentages for categorical variables.

To assess associations of the biomarkers with selected participant characteristics, we used multivariable general linear models to compare adjusted mean biomarker expression across categories (e.g., tertiles of dietary intakes) of the participant characteristics. All macronutrients were analyzed as their percentage contribution to total energy intake, and all micronutrients were analyzed as nutrient densities (e.g., mg of calcium/1,000 kcal of total energy intake). For any exposure-biomarker association, we considered biomarker staining batch, adenoma characteristics, and all remaining exposure variables as potential confounding variables. A priori, we elected to include biomarker staining batch and total energy intake in all models. Other confounders were exposures that were estimated to be at least modestly associated with a given biomarker via using forward model selection with a cutoff of p ≤ 0.4, followed by backward model selection of the resulting covariates with a cutoff of p ≤ 0.2. All final model covariates are listed in the Tables’ footnotes. As previously described (11, 14), we analyzed biomarker expression in whole crypts and in crypt functional zones, including the upper 40% of crypts (the canonical differentiation zone), and the lower 60% of crypts (the canonical proliferation zone). We also assessed differences in the balance of anti-proliferative APC relative to pro-proliferative β-catenin (calculated as an APC/β-catenin ratio), and of MSH2 relative to proliferation (calculated as a MSH2/mib-1 ratio) in whole crypts and the differentiation and proliferation zones of crypts. For perspective on differences in mean biomarker expression across participant characteristics categories, we calculated proportional mean differences as: (comparison group mean - reference group mean)/(reference group mean) x 100%.

We conducted all statistical analyses using SAS software Version 9.4 (SAS Institute, Cary, NC). We considered two-sided p-values ≤0.05 statistically significant. Since this was a pilot study to estimate the strengths and variability of hypothesized associations for further investigation in a larger observational study or randomized, controlled trials, we also noted participant characteristics for which estimated adjusted mean biomarker proportional differences between those in the highest relative to the lowest or referent participant characteristic category was ≥15%, and with a roughly dose-response or threshold pattern of the means across the categories (if >2 categories) and a p-value ≤0.15.

## Results

### Participant characteristics

Selected characteristics of the 104 study participants are summarized in **Table 1**. Participants’ ages ranged from 47 – 75 years (mean 59 years), 46% of participants were male, and 79% were white. Most (97%) participants had at least a high school education, 8% were current smokers, 38% reported high physical activity levels, 18% had an advanced adenoma (size ≥ 1.0 cm, villous component, or high-grade dysplasia), and 28% had multiple (>1) adenomas. Participants’ body mass indexes (BMI) ranged from 21.0 – 54.1 kg/m^2^ (mean 29.6 kg/m^2^), and their serum 25-OH-vitamin D concentrations ranged from 12.9 – 68.8 ng/mL (mean 24.1 ng/mL).

**Table 1:** Selected baseline characteristics of the study participants^a^ (*n* = 104).

Characteristics	Mean or proportion	SD	Range
**Demographics**			
Sex (%)			
Male	46.2		
Female	53.8		
Race (%)			
White	78.9		
Black	19.2		
Other	1.9		
Age (yrs.)	58.9	6.7	47 – 75
**Lifestyle**			
Currently smoke (%)	7.7		
Alcohol consumption (%)			
Low^b^	54.8		
High^b^	45.2		
Regularly^c^ take aspirin (%)	38.5		
Regularly^c^ take non-aspirin NSAID (%)	33.7		
Highest education level (%)			
High school	34.6		
College	36.5		
Graduate	26.0		
Physical activity level^d^ (%)			
Low	28.8		
Moderate	31.7		
High	38.5		
Body mass index (kg/m^2^)^e^	29.6	5.6	21.0 – 54.1
< 25	21.8		
25.0 – 29.9	41.6		
≥ 30.0	36.6		
**Dietary intake**			
Total energy (kcal/day)	1,349	763	630 – 2,936
Saturated fat (as % of total energy)	11.5	2.7	4.2 – 17.4
Total fat (as % of total energy)	36.3	7.4	17.8 – 58.5
Total^f^ calcium (mg/1,000 kcal)	425	161	170 – 1,085
Total^f^ vitamin E (mg/1,000 kcal)	5.6	1.9	2.6 – 15.6
Dietary fiber (g/1,000 kcal)	10.7	4.0	4.6 – 22.4
Total vegetables and fruit (servings/day)	4.6	2.2	0.7 – 12.5
Total meat (servings/day)	1.7	0.9	0.2 – 5.0
**Serum concentrations**			
25-OH vitamin D (ng/mL)	24.1	9.3	12.9 – 68.8
**Adenoma characteristics**			
With advanced adenoma^g^ (%)	17.8		
Multiple (> 1) adenomas (%)	27.7		

SD, standard deviation; NSAID, non-steroidal anti-inflammatory drug.

a A subset of colorectal adenoma patients participating in the Calcium/Vitamin D, Biomarkers and Colon Polyp Prevention Trial from the South Carolina and Georgia clinical centers.

b Low alcohol consumption is ≤ 0.8 drinks/day for men, 0 drinks/day for women; high alcohol consumption is > 0.8 drinks/day for men, > 0 drinks/day for women.

c Take at least once a week.

d From the short form International Physical Activity Questionnaire (IPAQ; publicly available at www.ipaq.ki.se).

e Categorized according to World Health Organization guidelines for normal, overweight, and obese.

f Dietary plus supplemental intake.

g Size ≥ 1.0 cm, villous component, or high-grade dysplasia.

### Biomarker expression, by participant characteristics

Proportional differences across categories of selected participant characteristics in adjusted mean APC, β-catenin, E-cadherin, and MSH2 expression in whole crypts and the differentiation and proliferation zones of crypts are presented in **Tables 2**–**5**. Representative images of whole crypts immunohistochemically processed and analyzed for APC, β-catenin, E-cadherin, and MSH2 expression are shown in **Figure 1**. In **Table 2**, we summarize the strongest findings for all of the biomarkers. The criteria for inclusion in this table were: estimated proportional mean differences in biomarker expression between the highest and lowest categories of the exposure variable ≥15%, plus an approximate dose-response or threshold pattern (if >2 categories) and a p-value ≤0.15. More comprehensive findings and exact values are provided in **Tables 3**–**5** and **Supplemental Tables 1–5**. Only the findings meeting these criteria for inclusion in **Table 2** are described in the remainder of the Results section below.

**Table 2:** Summary of adjusted mean differences^a^ in the expression of colon carcinogenesis pathway proteins in the normal-appearing colorectal mucosa of colorectal adenoma patients (*n* = 104) across categories of participant characteristics.

Participant characteristics	Biomarkers															
	**APC**			**β-catenin**			**APC/β-catenin** ^b^			**E-cadherin**			**MSH2**			**MSH2/mib-1** ^c^
** **	Whole crypt	Upper 40%^d^	Lower 60%^e^	Whole crypt	Upper 40%	Lower 60%	Whole crypt	Upper 40%	Lower 60%	Whole crypt	Upper 40%	Lower 60%	Whole crypt	Upper 40%	Lower 60%	Whole crypt
**Lifestyle, demographics**																
Age																
Female	↑**+**	↑**+**	↑**+**				↑**+**	↑**+**	↑**+**							
Take aspirin																(↓**+**)
Take non-aspirin NSAID																
Currently smoke																
Alcohol consumption		↓														
Physical activity																
Body mass index											(↑)			↓		
**Dietary intakes**																
Saturated fat																
Total fat																
Total^f^ vitamin E																
Total^f^ calcium																
Dietary fiber																
Meat				↑		↑	↓	↓	↓					↓		
Vegetables and fruit																
**Serum concentrations**																
25-OH vitamin D																

NSAID, non-steroidal anti-inflammatory drug.

a Mean differences across categories of participant characteristics assessed using multivariable general linear models containing the characteristic of interest, staining batch, and measured confounding variables (see **Tables 3**–**5** and **Supplemental Tables 1–5**). Criteria for inclusion in table: estimated proportional mean difference ≥ 15% plus a p-value ≤ 0.15 for the estimated difference, plus a dose-response or threshold association pattern (if > 2 categories). Up/down arrows indicate the direction (higher or lower, respectively) of the mean biomarker difference between a higher exposure category relative to the reference category. Arrows in brackets [e.g., (↑)] indicate that the direction of the difference was opposite that hypothesized. + Indicates a statistically significant (p ≤ 0.05) finding.

b APC expression divided by β-catenin expression in the whole crypt, upper 40% of crypts, and lower 60% of crypts.

c MSH2 expression divided by mib-1 (Ki-67 epitope) expression in the whole crypt.

d Biomarker expression in the upper 40% of the crypt (the canonical differentiation zone).

e Biomarker expression in the lower 60% of the crypt (the canonical proliferation zone).

f Total = dietary + supplemental.

**Table 3:** Comparisons of proportional differences in adjusted mean APC and β-catenin expression^a^ in the normal-appearing colorectal mucosa of sporadic colorectal adenoma patients (*n* = 104), by selected participant characteristics.

		APC						β-catenin					
Characteristics	*n*	Whole crypts, prop. diff. (%)	*p*	Upper 40% of crypts,^b^ prop. diff. (%)	*p*	Lower 60% of crypts,^c^ prop. diff. (%)	*p*	Whole crypts, prop. diff. (%)	*p*	Upper 40% of crypts,^b^ prop. diff. (%)	*p*	Lower 60% of crypts,^c^ prop. diff. (%)	*p*
**Age (yrs)**													
47 – 54	34	–		–		–		–		–		–	
55 – 62	35	8.6		12.4		5.3		2.2		-3.2		1.8	
63 – 75	35	7.1	0.62	15.0	0.42	0.1	0.87	5.4	0.79	10.0	0.65	2.9	0.93
**Sex**													
Male	48	–		–		–		–		–		–	
Female	56	322.9	< 0.01	296.7	< 0.01	399.1	< 0.01	22.8	0.29	4.2	0.91	33.5	0.14
**Regularly** ^d^ **take aspirin**													
No	64	–		–		–		–		–		–	
Yes	40	-1.8	0.80	-6.9	0.42	2.9	0.92	-0.5	0.93	0.2	0.99	-0.6	0.93
**Regularly** ^d^ **take other NSAID**													
No	69	–		–		–		–		–		–	
Yes	35	1.5	0.84	9.1	0.33	-4.4	0.62	-2.5	0.69	-11.0	0.31	-2.3	0.70
**Currently smoke**													
No	96	–		–		–		–		–		–	
Yes	8	-1.4	0.91	-5.2	0.74	2.8	0.86	8.8	0.42	10.5	0.61	7.4	0.50
**Alcohol consumption**													
Low^e^	57	–		–		–		–		–		–	
High^e^	47	-10.2	0.18	-15.9	0.08	-4.6	0.64	1.6	0.82	-4.5	0.73	2.5	0.73
**Physical activity**													
Low	30	–		–		–		–		–		–	
Moderate	38	-3.2		-7.3		1.0		-0.4		-4.6		-0.7	
High	27	-1.2	0.94	-6.3	0.77	4.3	0.93	-5.8	0.61	13.0	0.36	-5.9	0.61
**Body mass index (kg/m^2^)**													
< 25.0	22	–		–		–		–		–		–	
25.0 – 29.9	43	14.9		17.0		12.2		10.8		-14.5		11.1	
≥ 30	39	20.4	0.18	22.1	0.27	17.1	0.47	8.7	0.41	-8.6	0.53	8.7	0.39
**Total energy, tertiles**													
1	34	–		–		–		–		–		–	
2	35	-3.3		-5.9		-1.2		16.4		-8.6		15.0	
3	35	12.4	0.39	10.2	0.48	14.3	0.56	5.7	0.10	-17.7	0.36	5.0	0.14
**Saturated fat (% of total energy), tertiles**													
1	34	–		–		–		–		–		–	
2	35	2.0		-4.6		8.2		5.2		27.4		4.6	
3	35	-8.5	0.56	-4.2	0.90	-12.3	0.26	-8.0	0.27	5.2	0.14	-9.2	0.25
**Total fat (% of total energy), tertiles**													
1	34	–		–		–		–		–		–	
2	35	3.0		-1.0		6.5		-6.1		7.7		-5.3	
3	35	-3.4	0.78	2.8	0.94	-9.3	0.38	-12.4	0.19	-1.1	0.30	-12.6	0.18
**Total** ^f^ **vitamin E/1,000 kcal, tertiles**													
1	34	–		–		–		–		–		–	
2	35	16.5		18.6		13.7		-6.8		-3.3		-7.8	
3	35	14.1	0.25	11.0	0.33	16.9	0.43	-8.6	0.53	21.4	0.25	-8.3	0.49
**Total** ^f^ **calcium/1,000 kcal, tertiles**													
1	34	–		–		–		–		–		–	
2	35	-3.4		-5.3		-1.7		3.6		20.3		4.7	
3	35	-6.3	0.79	2.2	0.81	-14.1	0.41	7.6	0.64	-0.1	0.30	7.1	0.68
**Dietary fiber/1,000 kcal, tertiles**													
1	34	–		–		–		–		–		–	
2	35	4.2		-1.5		11.2		-5.5		24.5		-5.2	
3	35	15.5	0.51	-1.9	0.99	35.9	0.16	-4.8	0.78	10.6	0.34	-4.7	0.81
**Total meat intake, tertiles**													
1	34	–		–		–		–		–		–	
2	35	-14.9		-8.5		-20.2		20.5		-14.0		21.1	
3	35	-17.3	0.18	-18.4	0.39	-15.4	0.17	20.5	0.07	-3.3	0.53	19.6	0.06
**Total vegetables and fruit intake, tertiles**													
1	34	–		–		–		–		–		–	
2	35	-13.8		-18.1		-9.8		-3.0		17.4		-2.4	
3	35	-13.0	0.26	-13.5	0.22	-13.9	0.54	-2.0	0.93	6.4	0.54	0.0	0.93
**Serum 25-OH-vitamin D (ng/mL)**													
< 17.9	34	–		–		–		–		–		–	
17.9 – 26.9	35	-2.6		-6.6		2.1		-15.3		11.2		-16.1	
> 26.9	35	-2.1	0.70	1.9	0.73	-5.4	0.79	-4.3	0.07	-3.0	0.59	-5.2	0.05

NSAID, non-steroidal anti-inflammatory drug; prop. diff., proportional difference.

a Biomarker expression measured using automated immunohistochemistry with image analysis. Proportional differences in adjusted mean biomarker expression calculated as follows: first, adjusted mean differences in optical densities of biomarker labeling were calculated (see **Supplemental Tables 1** and **2** for exact means and 95% confidence intervals) using general linear models, adjusted for staining batch, total energy intake, and other confounding variables (for APC models: sex and intakes of total meat, dietary fiber/1,000 kcal of total energy intake, and total vegetables and fruit; for β-catenin models: total fat intake as a percentage of total energy intake); second, the proportional mean differences were calculated from the adjusted mean differences as (comparison group mean - reference group mean)/(reference group mean) x 100%.

b Biomarker expression in the upper 40% of the crypt (the canonical differentiation zone).

c Biomarker expression in the lower 60% of the crypt (the canonical proliferation zone).

d Take at least once a week.

e Low alcohol consumption is ≤ 0.8 drinks/day for men, 0 drinks/day for women; high alcohol consumption is > 0.8 drinks/day for men, > 0 drinks/day for women.

f Dietary plus supplemental intake.

**Table 4:** Comparisons of adjusted mean APC/β-catenin and MSH2/mib-1 expression^a^ in the normal-appearing colorectal mucosa of sporadic colorectal adenoma patients (*n* = 104), by selected participant characteristics.

	APC/β-catenin^b^							MSH2/mib-1^c^		
Characteristics	*n*	Whole crypts, prop. diff. (%)	*p*	Upper 40% of crypts,^d^ prop. diff. (%)	*p*	Lower 60% of crypts,^e^ prop. diff. (%)	*p*	Whole crypts, prop. diff. (%)		*p*
**Age (yrs)**										
47 – 54	34	–		–		–			–	
55 – 62	35	8.7		10.0		11.5			-1.9	
63 – 75	35	0.0	0.68	0.0	0.69	-3.8	0.75		0.0	0.98
**Sex**										
Male	48	–		–		–			–	
Female	56	191.7	0.01	210.0	0.02	207.7	0.03		-21.9	0.22
**Regularly** ^f^ **take aspirin**										
No	64	–		–		–			–	
Yes	40	-4.2	0.84	5.0	0.57	-13.8	0.34		-15.4	0.04
**Regularly** ^f^ **take other NSAID**										
No	69	–		–		–			–	
Yes	35	4.3	0.76	-4.8	0.61	16.7	0.30		-1.8	0.82
**Currently smoke**										
No	96	–		–		–			–	
Yes	8	-12.5	0.58	-4.8	0.78	-14.8	0.56		-11.7	0.43
**Alcohol consumption**										
Low^g^	57	–		–		–			–	
High^g^	47	-11.5	0.30	-13.0	0.39	-10.3	0.42		-14.8	0.12
**Physical activity**										
Low	30	–		–		–			–	
Moderate	38	4.3		5.0		0.0			-10.6	
High	27	0.0	0.99	5.0	0.93	-3.7	0.99		-4.7	0.55
**Body mass index (kg/m^2^)**										
< 25.0	22	–		–		–			–	
25.0 – 29.9	43	0.0		0.0		4.0			1.9	
≥ 30	39	13.6	0.62	10.0	0.69	16.0	0.73		-1.9	0.94
**Total energy, tertiles**										
1	34	–		–		–			–	
2	35	-16.7		-10.0		-24.1			-15.8	
3	35	12.5	0.09	20.0	0.19	3.4	0.11		-1.2	0.18
**Saturated fat (% of total energy), tertiles**										
1	34	–		–		–			–	
2	35	-8.3		5.0		-17.2			16.4	
3	35	0.0	0.79	5.0	0.96	-3.4	0.36		15.1	0.25
**Total fat (% of total energy), tertiles**										
1	34	–		–		–			–	
2	35	14.3		10.0		12.5			7.2	
3	35	19.0	0.75	0.0	0.78	20.8	0.49		8.5	0.66
**Total** ^h^ **vitamin E/1,000 kcal, tertiles**										
1	34	–		–		–			–	
2	35	19.0		16.7		25.0			-6.1	
3	35	14.3	0.39	27.8	0.41	4.2	0.35		-1.2	0.81
**Total** ^h^ **calcium/1,000 kcal, tertiles**										
1	34	–		–		–			–	
2	35	-8.0		-13.0		0.0			-6.6	
3	35	-8.0	0.73	-17.4	0.53	-3.7	0.98		-4.2	0.80
**Dietary fiber/1,000 kcal, tertiles**										
1	34	–		–		–			–	
2	35	-4.0		5.0		12.0			-6.3	
3	35	-16.0	0.82	5.0	0.96	4.0	0.78		-19.3	0.24
**Total meat intake, tertiles**										
1	34	–		–		–			–	
2	35	-27.6		-28.0		-26.5			11.2	
3	35	-27.6	0.11	-20.0	0.15	-35.3	0.08		5.9	0.63
**Total vegetables and fruit intake, tertiles**										
1	34	–		–		–			–	
2	35	-8.0		-9.1		-7.1			-4.1	
3	35	-12.0	0.76	-9.1	0.88	-7.1	0.87		-15.1	0.32
**Serum 25-OH-vitamin D (ng/mL)**										
< 17.9	34	–		–		–			–	
17.9 – 26.9	35	8.7		21.1		0.0			17.4	
> 26.9	35	-4.3	0.74	0.0	0.45	-3.7	0.97		6.7	0.30

NSAID, non-steroidal anti-inflammatory drug; prop. diff., proportional difference.

a Biomarker expression measured using automated immunohistochemistry with image analysis. Proportional differences in adjusted mean biomarker expression calculated as follows: first, adjusted mean differences in optical densities of biomarker labeling were calculated (see **Supplemental Table 3** for exact means and 95% confidence intervals) using general linear models, adjusted for staining batch, total energy intake, and other confounding variables (for APC/β-catenin models: sex and intakes of total meat, dietary fiber/1,000 kcal of total energy intake, and total vegetables and fruit; for MSH2/mib-1 models: aspirin use and total calcium intake/1,000 kcal of total energy intake); second, the proportional mean differences were calculated from the adjusted mean differences as (comparison group mean - reference group mean)/(reference group mean) x 100%.

b APC expression divided by β-catenin expression.

c MSH2 expression divided by mib-1 expression.

d Biomarker expression in the upper 40% of the crypt (the canonical differentiation zone).

e Biomarker expression in the lower 60% of the crypt (the canonical proliferation zone).

f Take at least once a week.

g Low alcohol consumption is ≤ 0.8 drinks/day for men, 0 drinks/day for women; high alcohol consumption is > 0.8 drinks/day for men, > 0 drinks/day for women.

h Dietary plus supplemental.

**Table 5:** Comparisons of proportional differences in adjusted mean E-cadherin and MSH2 expression^a^ in the normal-appearing colorectal mucosa of sporadic colorectal adenoma patients (*n* = 104), by selected participant characteristics.

		E-cadherin						MSH2					
Characteristics	*n*	Whole crypts, prop. diff. (%)	*p*	Upper 40% of crypts,^b^ prop. diff. (%)	*p*	Lower 60% of crypts,^c^ prop. diff. (%)	*P*	Whole crypts, prop. diff. (%)	*p*	Upper 40% of crypts,^b^ prop. diff. (%)	*p*	Lower 60% of crypts,^c^ prop. diff. (%)	*p*
**Age (yrs)**													
47 – 54	34	–		–		–		–		–		–	
55 – 62	35	2.8		-36.5		3.7		0.9		-31.4		2.0	
63 – 75	35	-4.2	0.75	-35.5	0.29	-5.7	0.63	1.3	0.99	165.3	0.51	-0.3	0.96
**Sex**													
Male	48	–		–		–		–		–		–	
Female	56	6.9	0.72	127.8	0.27	9.3	0.64	-6.4	0.72	10.1	0.97	-10.1	0.55
**Regularly** ^d^ **take aspirin**													
No	64	–		–		–		–		–		–	
Yes	40	-2.1	0.78	-4.1	0.88	-2.8	0.72	-14.3	0.04	43.0	0.71	-12.6	0.06
**Regularly** ^d^ **take other NSAID**													
No	69	–		–		–		–		–		–	
Yes	35	-0.7	0.92	36.4	0.31	-0.4	0.97	-5.4	0.45	-63.6	0.25	-5.1	0.46
**Currently smoke**													
No	96	–		–		–		–		–		–	
Yes	8	-7.9	0.56	53.4	0.31	-9.7	0.49	1.2	0.93	-173.6	0.26	-1.4	0.92
**Alcohol consumption**													
Low^e^	57												
High^e^	47	-3.9	0.63	-3.2	0.92	-3.5	0.68	-8.3	0.31	6.8	0.95	-7.1	0.38
**Physical activity**													
Low	30	–		–		–		–		–		–	
Moderate	38	-2.1		42.6		-3.7		-9.3		-68.1		7.1	
High	27	-10.1	0.43	-44.6	0.01	-12.1	0.36	3.1	0.74	70.3	0.46	3.1	0.74
**Body mass index (kg/m^2^)**													
< 25.0	22	–		–		–		–		–		–	
25.0 – 29.9	43	2.4		133.9		3.0		3.3		-73.2		1.5	
≥ 30	39	4.2	0.92	128.0	0.15	5.5	0.87	-1.2	0.84	-87.5	0.15	-3.3	0.83
**Total energy, tertiles**													
1	34	–		–		–		–		–		–	
2	35	-9.2		51.5		-10.0		2.7		-93.2		5.0	
3	35	4.8	0.26	45.3	0.73	0.3	0.42	5.8	0.82	-34.2	0.36	8.1	0.06
**Saturated fat (% of total energy), tertiles**													
1	34	–		–		–		–		–		–	
2	35	17.8		22.5		18.7		-2.8		121.6		-2.3	
3	35	2.8	0.12	-0.4	0.77	4.0	0.13	-5.8	0.83	91.1	0.81	-7.8	0.69
**Total fat (% of total energy), tertiles**													
1	34	–		–		–		–		–		–	
2	35	10.1		-12.8		12.5		-1.2		277.8		-1.7	
3	35	-0.2	0.44	5.0	0.86	1.7	0.38	-1.4	0.99	-54.5	0.22	-2.5	0.96
**Total** ^f^ **vitamin E/1,000 kcal, tertiles**													
1	34	–		–		–		–		–		–	
2	35	-5.5		13.9		-8.8		-6.7		55.7		-6.5	
3	35	-5.8	0.80	-24.8	0.49	-8.0	0.62	-2.1	0.73	179.9	0.72	-3.3	0.75
**Total** ^f^ **calcium/1,000 kcal, tertiles**													
1	34	–		–		–		–		–		–	
2	35	-7.0		0.1		-7.2		-16.9		85.2		-14.2	
3	35	7.2	0.36	-4.7	0.99	6.2	0.44	-7.7	0.14	6.7	0.86	-9.0	0.11
**Dietary fiber/1,000 kcal, tertiles**													
1	34	–		–		–		–		–		–	
2	35	-1.5		-44.8		-3.4		-10.4		138.5		-8.3	
3	35	6.1	0.79	81.1	0.02	0.7	0.91	1.7	0.32	-115.0	0.15	2.4	0.43
**Total meat intake, tertiles**													
1	34	–		–		–		–		–		–	
2	35	-9.9		101.4		-5.9		0.4		-108.2		1.8	
3	35	-3.6	0.53	44.5	0.16	-2.6	0.82	-11.5	0.45	-64.4	0.10	-10.8	0.41
**Total vegetables and fruit intake, tertiles**													
1	34	–		–		–		–		–		–	
2	35	16.7		-53.1		15.8		3.4		482.0		2.7	
3	35	16.2	0.22	-14.3	0.12	19.7	0.20	-4.8	0.66	-64.4	0.13	-4.3	0.73
**Serum 25-OH-vitamin D (ng/mL)**													
< 17.9	34	–		–		–		–		–		–	
17.9 – 26.9	35	-9.7		-49.3		-11.5		-4.2		783.3		-3.2	
> 26.9	35	-0.7	0.50	0.0	0.16	-1.3	0.42	6.5	0.54	289.5	0.37	5.7	0.64

NSAID, non-steroidal anti-inflammatory drug; prop. diff., proportional difference.

a Biomarker expression measured using automated immunohistochemistry with image analysis. Proportional differences in adjusted mean biomarker expression calculated as follows: first, adjusted mean differences in optical densities of biomarker labeling were calculated (see **Supplemental Tables 4** and **5** for exact means and 95% confidence intervals) using general linear models, adjusted for staining batch, total energy intake, and other confounding variables (for E-cadherin models: total vegetables and fruit intake; for MSH2 models: aspirin use and total calcium intake/1,000 kcal of total energy intake); second, the proportional mean differences were calculated from the adjusted mean differences as (comparison group mean - reference group mean)/(reference group mean) x 100%.

b Biomarker expression in the upper 40% of the crypt (the canonical differentiation zone).

c Biomarker expression in the lower 60% of the crypt (the canonical proliferation zone).

d Take at least once a week.

e Low alcohol consumption is ≤ 0.8 drinks/day for men, 0 drinks/day for women; high alcohol consumption is > 0.8 drinks/day for men, > 0 drinks/day for women.

f Dietary plus supplemental intake.

### APC

As shown in **Table 3**, the mean adjusted APC expression among women relative to men was, proportionately, 322.9% higher (p<0.01) in whole crypts, 296.7% higher (p<0.01) in the differentiation zone of crypts, and 399.1% higher (p<0.01) in the proliferation zone of crypts. The mean adjusted APC expression among those with higher alcohol consumption relative to those with lower alcohol consumption was estimated to be 15.9% lower (p=0.08) in the differentiation zone of crypts.

### β-catenin

As shown in **Table 3**, the mean adjusted β-catenin expression among participants in the highest relative to those in the lowest total meat consumption tertile was estimated to be 20.5% higher (p=0.07) in whole crypts and 19.6% higher (p=0.06) in the proliferation zone of crypts.

### APC/β-catenin ratio

As shown in **Table 4**, the mean adjusted ratio of APC expression to β-catenin expression (APC/β-catenin ratio) among women relative to men was 191.7% higher (p=0.01) in whole crypts, 210.0% higher (p=0.02) in the differentiation zone of crypts, and 207.7% higher (p=0.03) in the proliferation zone of crypts. The APC/β-catenin ratio among participants in the highest relative to the lowest total meat consumption tertile was estimated to be 27.6% lower (p=0.11) in whole crypts, 20.0% lower (p=0.15) in the differentiation zone of crypts, and 35.3% lower (p=0.08) in the proliferation zone of crypts.

### E-cadherin

As shown in **Table 5**, the mean adjusted E-cadherin expression among participants in the highest relative to those in the lowest BMI tertile was estimated to be 128.0% higher (p=0.15) in the differentiation zone of crypts. The observed association for BMI was in the opposite direction hypothesized.

### MSH2

As shown in **Table 5**, the mean adjusted MSH2 expression among participants in the highest relative to those in the lowest BMI tertile was estimated to be 87.5% lower (p=0.15) in the differentiation zone of crypts. The mean adjusted MSH2 expression among participants in the highest relative to those in the lowest total meat consumption tertile was estimated to be 64.4% lower (p=0.10) in the differentiation zone of crypts.

### MSH2/mib-1 ratio

As shown in **Table 4**, the ratio of mean adjusted MSH2 to mib-1 expression (MSH2/mib-1 ratio) among participants who regularly took aspirin at least once a week or more relative to those who did not was estimated to be 15.4% lower (p=0.04) in whole crypts, although the direction of this association was opposite to that hypothesized.

## Discussion

Our findings suggest that sex, alcohol consumption, adiposity, aspirin, and meat consumption each may be associated with biomarkers of colorectal carcinogenesis pathway proteins in the normal-appearing colorectal mucosa of sporadic colorectal adenoma patients. Our findings, although cross-sectional, suggest that the exposures may affect colorectal carcinogenesis pathways in the colorectal epithelium and thus, risk for colorectal neoplasms, which supports further investigation in larger observational studies and randomized, controlled trials.

More specifically, our results suggest that, based on their estimated associations with APC, β-catenin, and E-cadherin expression, being male and greater alcohol and meat consumption may be associated with a colorectal mucosa at higher risk for colorectal carcinogenesis through the Wnt/APC colon carcinogenesis pathway. The findings for men included substantial, statistically significant higher expression of anti-proliferative APC alone and in its balance with pro-proliferative β-catenin expression in whole crypts and in the crypt differentiation and proliferation zones (upper 40% and lower 60% of crypts, respectively). Similarly, the findings for total meat consumption included higher estimated pro-proliferative β-catenin expression in whole crypts and the crypt proliferation zone, and lower APC relative to β-catenin expression (i.e., a more pro-proliferative balance) in whole crypts and in the crypt differentiation and proliferation zones. The findings for alcohol consumption were limited to lower APC expression in the crypt differentiation zone.

However, our estimated association of BMI with E-cadherin expression was opposite to that we hypothesized. A higher BMI was estimated to be associated with higher E-cadherin expression in the differentiation zone of crypts (although not in whole crypts or in the proliferation zone of crypts). The reasons for this finding are unclear but could include unknown uncontrolled confounding and chance; it is also possible that some exposures may affect some biomarkers in ways that may increase risk (e.g., a higher BMI could decrease MSH2), and others in ways that may decrease risk (e.g., a higher BMI could increase E-cadherin).

Our findings also suggest that, based on their associations with the expression of the MSH2 DNA MMR protein, higher BMI and total meat consumption may be associated with a colorectal mucosa at higher risk for colorectal carcinogenesis through impaired DNA MMR. It is noted that the findings for BMI and meat consumption in relation to MSH2 expression were limited to the crypt differentiation zone. However, our findings for aspirin use were opposite to those hypothesized. Regularly taking aspirin (but not other non-steroidal anti-inflammatory drugs [NSAIDs]) was associated with lower expression of MSH2 relative to proliferation (the MSH2/mib-1 ratio), although only in whole crypts, and MSH2 expression alone did not differ according to aspirin use. The findings for aspirin being opposite to those hypothesized and different from those for other NSAIDs are unclear but suggest chance and/or unknown uncontrolled confounding.

Cellular mechanisms for associations of various dietary and medical characteristics with APC, β-catenin, E-cadherin, and MSH2 have been reported. Meat, especially red meat, contains high levels of heme, an iron-porphyrin metalloprotein. In rats, heme consumption induced lipid peroxidation in the colon leading to cell surface damage and APC mutation (15). Alcohol increased APC mutation and intestinal tumorigenesis in mice (16). Larger BMI was also previously associated with aberrant expression of MMR proteins, including MSH2 (17). Finally, while CRC incidence is lower among women, which is consistent with our finding that women had higher APC expression and a higher APC/β-catenin expression ratio, the mechanism behind such differences in protein expression is not well understood (18).

Whether calcium and vitamin D exposures affect the aforementioned biomarkers was investigated in two small, randomized, controlled trials (10–12, 14) and one observational study (8, 9). In contrast to the present observational study in which there was no substantial evidence of associations of serum 25-OH-vitamin D concentrations with the biomarkers reported herein, in both trials, vitamin D3 supplementation increased APC or the APC/β-catenin ratio and MSH2 or the MSH2/mib-1 ratio. In one of the two trials (the one not related to the present study population (10, 12)), supplemental vitamin D3 also increased E-cadherin expression. In that same trial, supplemental calcium (2.0 g/day) also increased the APC/β-catenin ratio and MSH2 expression. However, in the other trial (11, 14), which was conducted with the same participants as in the present study, supplemental calcium (1.2 g/day) had no apparent effect on the biomarkers. In a cross-sectional analysis of an outpatient, elective colonoscopy population (8, 9), higher serum 25-OH-vitamin D concentrations were associated with a higher APC/β-catenin ratio, but were not associated with E-cadherin or MSH2 expression (an MSH2/mib-1 ratio was not investigated). Total calcium intakes were not substantially or statistically significantly associated with APC, β-catenin, the APC/β-catenin ratio, or MSH2 expression.

Likely reasons for the discrepancies in findings between the present study and the previous investigations of calcium, vitamin D and the biomarkers reported herein are: 1) in the present study, prospective participants with serum 25-OH-vitamin D concentrations <12 or >90 ng/mL were excluded from participation, thus restricting the range of vitamin D exposures for the cross-sectional analyses, which would tend to yield attenuated associations; and 2) in the clinical trials, those randomized to supplemental vitamin D or calcium received much higher amounts of those agents than did those randomized to placebo, which would more likely yield positive findings if there is a true effect. Taken together, previous literature supports that higher vitamin D exposures may increase the APC/β-catenin ratio and E-cadherin and MSH2 expression, and higher calcium intakes may also increase the APC/β-catenin ratio and MSH2 expression in the normal-appearing colorectal mucosa. However, the present and previous studies taken together do not appear to support that higher calcium exposures substantially affect E-cadherin expression.

To the best of our knowledge, only one reported study, the aforementioned cross-sectional analysis of an outpatient, elective colonoscopy population (8, 9), investigated associations of other exposures in humans with the expression of the biomarkers investigated in the present study. In cross-sectional analyses of subsets of that study population, APC/β-catenin scores were lower among men and those with higher total fat and processed meat intakes (a variable not available for the present study). β-catenin expression alone was higher among alcohol consumers and those with higher waist to hip ratios (although BMI was measured in the present study, waist to hip ratios were not). MSH2 expression was higher among those who took an NSAID (aspirin or other NSAID). However, contrary to hypotheses, the APC/β-catenin scores and APC expression alone were lower among those with higher physical activity and those who regularly took a NSAID, and β-catenin expression was higher among those who regularly took a NSAID. The findings between the previous and present study are generally consistent with each other in relation to sex, alcohol consumption, meat intakes, and adiposity, but are dissimilar in relation to physical activity and NSAID use. The studies also were consistent with one another in relation to estimated null associations of various other participant characteristics with the various biomarkers. The reasons for the few discrepancies between the findings of the two studies are unclear but may involve differences in dietary assessment (the previous study used a comprehensive Willett FFQ, whereas the present study used a more limited Block Brief 2000 FFQ), and differences in study populations. The colonoscopy population comprised both patients with and without colorectal adenoma, whereas our study population comprised only colorectal adenoma patients participating in a long-term chemoprevention trial.

Our study addresses the goal of our research group, which is to identify, develop, and apply modifiable, pre-neoplastic, phenotypic biomarkers of risk for colorectal neoplasms, analogous to lipid profiles for ischemic heart disease (19). Such biomarkers can be used: 1) to increase understanding of relevant CRC etiologic mechanisms in humans; 2) to identify in observational studies, such as the one reported herein, exposures that potentially may modulate these mechanisms; 3) as endpoints in clinical trials to assess the potential efficacy, optimum dose, and potential long-term safety of interventions (identified in part via use 2); and 4) to help clinically assess and manage CRC risk. An example of the latter is that they could be used in conjunction with a baseline colonoscopy to personalize colonoscopy or other CRC screening intervals. Another example is that, if correlates of the tissue markers can be identified in easy to procure surrogate fluids, such as blood or urine, the surrogate measures could be used for managing CRC risk in a primary care setting as one would for ischemic heart disease prevention (19).

Our study had several strengths and limitations. A primary limitation was the small sample size, which limited our ability to detect associations or to conduct stratified analyses. However, the study was a pilot study to estimate the strengths and variability of potential associations of participant characteristics with the selected biomarkers. To this end, we estimated several associations that were 1) consistent with hypotheses, and 2) of sufficient magnitude and feasible variability, to support them as candidates for further investigation in a larger observational study and/or in randomized, controlled trials. We measured biomarkers only in rectal biopsies, so possible exposure-biomarker associations at other colon sites remain unknown. Our study was restricted to sporadic colorectal adenoma patients—all of whom were participating in a long-term, randomized, controlled chemoprevention trial—so, our findings may not be generalizable to other populations. Other limitations included the observational, cross-sectional design, which precludes assessing cause-effect, and that FFQs have known limitations (e.g., recall error and limited food and food preparation choices), which may yield attenuated associations. Our measurement methodology is semiquantitative, and cannot be strictly calibrated to standards; however, all procedures were done according to strict protocols across all study participants and our measurements were highly reliable, allowing biomarker values among participants and exposures to be validly ranked relative to one another. Finally, our measurement technology did not allow us to evaluate β-catenin sub-cellular localization; however, findings from a case-control study (8) suggested that total β-catenin expression in the normal colorectal mucosa was greater in sporadic colorectal adenoma cases relative to that in normal controls. Study strengths included: 1) the comprehensive assessment of multiple dietary, lifestyle, demographic, and medical factors, and 2) the automated immunostaining and novel image analysis software, which enabled quantification of crypt biomarker distributions and high biomarker scoring reliability.

In conclusion, the results of this pilot, cross-sectional study, taken together with previous literature, support that being male, higher adiposity, and higher alcohol and meat consumption each may be unfavorably associated with biomarkers of colorectal carcinogenesis pathways in the normal-appearing colorectal mucosa of sporadic colorectal adenoma patients. These associations, although preliminary and cross-sectional, suggest that various exposures may affect colorectal carcinogenesis pathways in the colorectal epithelium, and thus risk for colorectal neoplasms, and so support further investigation in larger observational studies and/or in randomized controlled trials. Our findings also support further study of the use of APC, β-catenin, E-cadherin, MSH2, and APC/β-catenin and MSH2/mib-1 ratios in the normal-appearing rectal mucosa, as potentially modifiable, pre-neoplastic markers of risk for colorectal neoplasms.

## Data availability statement

The raw data supporting the conclusions of this article will be made available by the authors, without undue reservation.

## Ethics statement

The studies involving human participants were reviewed and approved by Emory University Institutional Review Board. The patients/participants provided their written informed consent to participate in this study.

## Author contributions

TS conducted the analyses and was the primary manuscript writer. RR, MS, and EB contributed to data collection and manuscript review. RB led the overall study, including data collection, data analysis and interpretation, and reviewing and editing the manuscript. All authors read and approved the final manuscript.

## Funding

National Cancer Institute, National Institutes of Health (R01 CA114456 to RMB and R01 CA098286 to John A. Baron); Georgia Cancer Coalition Distinguished Scholar award (to RB); the Anne and Wilson P. Franklin Foundation (to RB).

## Acknowledgments

A preliminary version of some of the contents of this manuscript was previously included in an Emory University MPH in Epidemiology thesis, which appeared online (https://etd.library.emory.edu/concern/etds/ws859g73z?locale=en).

## Conflict of interest

Author MS was employed by Consultants in Gastroenterology.

The remaining authors declare that the research was conducted in the absence of any commercial or financial relationships that could be construed as a potential conflict of interest.

## Publisher’s note

All claims expressed in this article are solely those of the authors and do not necessarily represent those of their affiliated organizations, or those of the publisher, the editors and the reviewers. Any product that may be evaluated in this article, or claim that may be made by its manufacturer, is not guaranteed or endorsed by the publisher.

## Abbreviations

CRC, colorectal cancer; MMR, mismatch repair; IRB, institutional review board; FFQ, food frequency questionnaire; BMI, body mass index; NSAID, non-steroidal anti-inflammatory drug.

## References

[1] Edwards BK Ward E Kohler BA Eheman C Zauber AG Anderson RN. Annual report to the nation on the status of cancer, 1975-2006, featuring colorectal cancer trends and impact of interventions (risk factors, screening, and treatment) to reduce future rates. Cancer (2010) 116(3):544–73. doi: 10.1002/cncr.24760 PMC3619726 19998273

[2] American Cancer Society. Cancer Facts & Figures 2015. Atlanta: American Cancer Society (2015).

[3] Aleksandrova K Pischon T Jenab M Bueno-de-Mesquita HB Fedirko V Norat T. Combined impact of healthy lifestyle factors on colorectal cancer: a large European cohort study. BMC Med (2014) 12:168. doi: 10.1186/s12916-014-0168-4 25319089 PMC4192278

[4] Dowty JG Win AK Buchanan DD Lindor NM Macrae FA Clendenning M. Cancer risks for MLH1 and MSH2 mutation carriers. Hum Mutat (2013) 34(3):490–7. doi: 10.1002/humu.22262 PMC3887142 23255516

[5] Kwong LN Dove WF. APC and its modifiers in colon cancer. Adv Exp Med Biol (2009) 656:85–106. doi: 10.1007/978-1-4419-1145-2_8 19928355 PMC3754875

[6] Boland CR Goel A. Microsatellite instability in colorectal cancer. Gastroenterology (2010) 138(6):2073–87.e3. doi: 10.1053/j.gastro.2009.12.064 20420947 PMC3037515

[7] Komiya Y Habas R. Wnt signal transduction pathways. Organogenesis (2008) 4(2):68–75. doi: 10.4161/org.4.2.5851 19279717 PMC2634250

[8] Ahearn TU Shaukat A Flanders WD Seabrook ME Bostick RM. Markers of the APC/beta-catenin signaling pathway as potential treatable, preneoplastic biomarkers of risk for colorectal neoplasms. Cancer Epidemiol Biomarkers Prev (2012) 21(6):969–79. doi: 10.1158/1055-9965.EPI-12-0126 22539608

[9] Sidelnikov E Bostick RM Flanders WD Long Q Seabrook ME. Colorectal mucosal expression of MSH2 as a potential biomarker of risk for colorectal neoplasms. Cancer Epidemiol Biomarkers Prev (2009) 18(11):2965–73. doi: 10.1158/1055-9965.EPI-09-0519 19861524

[10] Ahearn TU Shaukat A Flanders WD Rutherford RE Bostick RM. A randomized clinical trial of the effects of supplemental calcium and vitamin D3 on the APC/beta-catenin pathway in the normal mucosa of colorectal adenoma patients. Cancer Prev Res (Phila) (2012) 5(10):1247–56. doi: 10.1158/1940-6207.CAPR-12-0292 PMC3466388 22964475

[11] Liu S Barry EL Baron JA Rutherford RE Seabrook ME Bostick RM. Effects of supplemental calcium and vitamin d on the APC/beta-catenin pathway in the normal colorectal mucosa of colorectal adenoma patients. Mol Carcinog (2017) 56(2):412–24. doi: 10.1002/mc.22504 PMC5586148 27254743

[12] Sidelnikov E Bostick RM Flanders WD Long Q Fedirko V Shaukat A. Effects of calcium and vitamin d on MLH1 and MSH2 expression in rectal mucosa of sporadic colorectal adenoma patients. Cancer Epidemiol Biomarkers Prev (2010) 19(4):1022–32. doi: 10.1158/1055-9965.EPI-09-0526 PMC3513334 20332274

[13] Baron JA Barry EL Mott LA Rees JR Sandler RS Snover DC. A trial of calcium and vitamin d for the prevention of colorectal adenomas. N Engl J Med (2015) 373(16):1519–30. doi: 10.1056/NEJMoa1500409 PMC4643064 26465985

[14] Kwan AK Um CY Rutherford RE Seabrook ME Barry EL Fedirko V. Effects of vitamin d and calcium on expression of MSH2 and transforming growth factors in normal-appearing colorectal mucosa of sporadic colorectal adenoma patients: A randomized clinical trial. Mol Carcinog (2019) 58(4):511–23. doi: 10.1002/mc.22945 30499618

[15] Bastide NM Chenni F Audebert M Santarelli RL Tache S Naud N. A central role for heme iron in colon carcinogenesis associated with red meat intake. Cancer Res (2015) 75(5):870–9. doi: 10.1158/0008-5472.CAN-14-2554 25592152

[16] Na HK Lee JY. Molecular basis of alcohol-related gastric and colon cancer. Int J Mol Sci (2017) 18(6):1116. doi: 10.3390/ijms18061116 28538665 PMC5485940

[17] Sun Z Yu X Wang H Zhang S Zhao Z Xu R. Clinical significance of mismatch repair gene expression in sporadic colorectal cancer. Exp Ther Med (2014) 8(5):1416–22. doi: 10.3892/etm.2014.1927 PMC4186363 25289032

[18] White A Ironmonger L Steele RJC Ormiston-Smith N Crawford C Seims A. A review of sex-related differences in colorectal cancer incidence, screening uptake, routes to diagnosis, cancer stage and survival in the UK. BMC Cancer (2018) 18(1):906. doi: 10.1186/s12885-018-4786-7 30236083 PMC6149054

[19] Bostick RM. Effects of supplemental vitamin d and calcium on normal colon tissue and circulating biomarkers of risk for colorectal neoplasms. J Steroid Biochem Mol Biol (2015) 148:86–95. doi: 10.1016/j.jsbmb.2015.01.010 25597952 PMC4389892

