# Supplementary material for: Associations of dietary, lifestyle, and other participant characteristics with APC, β-catenin, E-cadherin, and MSH2 expression in the normal mucosa of sporadic colorectal adenoma patients
Source: Front Gastroenterol (Lausanne). 2022 Nov 7;1:889925. doi: 10.3389/fgstr.2022.889925 (PMC12952325; doi:10.3389/fgstr.2022.889925)
Supplement: Supplementary file 1 [file DataSheet_1.docx]

**Supplementary Materials**

**Supplemental Table 1.** Comparisons^a^ of adjusted mean APC expression^b^ in the normal-appearing colorectal mucosa of sporadic colorectal adenoma patients (*n* = 104), by selected participant characteristics.

| **Characteristics** | ***n*** | **Whole crypts, mean (OD)** | **95% CI** | **Prop. diff.**^c^ **(%)** | ***p*** | **Upper 40% of crypts, mean (OD)** | **95% CI** | **Prop. diff.**^c^ **(%)** | ***p*** | **Lower 60% of crypts, mean (OD)** | **95% CI** | **Prop. diff**^c^ **(%)** | ***p*** |
| --- | --- | --- | --- | --- | --- | --- | --- | --- | --- | --- | --- | --- | --- |
| **Age (yrs)** |  |  |  |  |  |  |  |  |  |  |  |  |  |
| 47 – 54 | 34 | 2,383 | (2,078, 2,689) | - |  | 929 | (778, 1,079) | - |  | 1,324 | (1,257, 1,570) | - |  |
| 55 – 62 | 35 | 2,589 | (2,290, 2,888) | 8.6 |  | 1,044 | (896, 1,191) | 12.4 |  | 1,394 | (1,167, 1,578) | 5.3 |  |
| 63 – 75 | 35 | 2,553 | (2,250, 2,856) | 7.1 | 0.62 | 1,068 | (919, 1,218) | 15.0 | 0.42 | 1,325 | (1,101, 1,518) | 0.1 | 0.87 |
| **Sex** |  |  |  |  |  |  |  |  |  |  |  |  |  |
| Male | 48 | 917 | (206, 1,629) | - |  | 391 | (39, 744) | - |  | 428 | (-52, 925) | - |  |
| Female | 56 | 3,878 | (3,263, 4,492) | 322.9 | <0.01 | 1,551 | (1,247, 1,856) | 296.7 | <0.01 | 2,136 | (1,706, 2,551) | 399.1 | <0.01 |
| **Regularly**^d^ **take aspirin** |  |  |  |  |  |  |  |  |  |  |  |  |  |
| No | 64 | 2,540 | (2,270, 2,810) | - |  | 1,061 | (927, 1,194) | - |  | 1,324 | (1,155, 1,525) | - |  |
| Yes | 40 | 2,494 | (2,288, 2,699) | -1.8 | 0.80 | 988 | (886, 1,089) | -6.9 | 0.42 | 1,363 | (1,212, 1,494) | 2.9 | 0.92 |
| **Regularly**^d^ **take other NSAID** |  |  |  |  |  |  |  |  |  |  |  |  |  |
| No | 69 | 2,487 | (2,204, 2,770) | - |  | 958 | (819, 1,097) | - |  | 1,388 | (1,192, 1,580) | - |  |
| Yes | 35 | 2,524 | (2,330, 2,717) | 1.5 | 0.84 | 1,045 | (950, 1,141) | 9.1 | 0.33 | 1,327 | (1,195, 1,461) | -4.4 | 0.62 |
| **Currently smoke** |  |  |  |  |  |  |  |  |  |  |  |  |  |
| No | 96 | 2,514 | (2,355, 2,673) | - |  | 1,020 | (941, 1,099) | - |  | 1,345 | (1,237, 1,453) | - |  |
| Yes | 8 | 2,478 | (1,859, 3,097) | -1.4 | 0.91 | 967 | (661, 1,273) | -5.2 | 0.74 | 1,383 | (964, 1,803) | 2.8 | 0.86 |
| **Alcohol consumption** |  |  |  |  |  |  |  |  |  |  |  |  |  |
| Low^e^ | 57 | 2,632 | (2,400,2,865) | - |  | 1,095 | (980, 1,209) | - |  | 1,376 | (1,217, 1,536) | - |  |
| High^e^ | 47 | 2,364 | (2,101, 2,628) | -10.2 | 0.18 | 921 | (791, 1,050) | -15.9 | 0.08 | 1,313 | (1,133, 1,493) | -4.6 | 0.64 |
| **Physical activity** |  |  |  |  |  |  |  |  |  |  |  |  |  |
| Low | 30 | 2,538 | (2,212, 2,863) | - |  | 1,064 | (903, 1,225) | - |  | 1,313 | (1,093, 1,534) | - |  |
| Moderate | 38 | 2,456 | (2,159, 2,754) | -3.2 |  | 986 | (839, 1,133) | -7.3 |  | 1,326 | (1,124, 1,527) | 1.0 |  |
| High | 27 | 2,507 | (2,232, 2,782) | -1.2 | 0.94 | 997 | (861, 1,132) | -6.3 | 0.77 | 1,369 | (1,183, 1,555) | 4.3 | 0.93 |
| **Body mass index (kg/m^2^)** |  |  |  |  |  |  |  |  |  |  |  |  |  |
| < 25.0 | 22 | 2,207 | (1,837, 2,576) | - |  | 881 | (697, 1,065) | - |  | 1,209 | (955, 1,463) | - |  |
| 25.0 – 29.9 | 43 | 2,535 | (2,282, 2,789) | 14.9 |  | 1,031 | (905, 1,157) | 17.0 |  | 1,357 | (1,183, 1,531) | 12.2 |  |
| ≥ 30 | 39 | 2,657 | (2,380, 2,933) | 20.4 | 0.18 | 1,076 | (938, 1,213) | 22.1 | 0.27 | 1,416 | (1,226, 1,606) | 17.1 | 0.47 |
| **Total energy, tertiles** |  |  |  |  |  |  |  |  |  |  |  |  |  |
| 1 | 34 | 2,438 | (2,052, 2,823) | - |  | 1,002 | (811, 1,193) | - |  | 1,291 | (1,033, 1,549) | - |  |
| 2 | 35 | 2,357 | (2,055, 2,658) | -3.3 |  | 943 | (794, 1,092) | -5.9 |  | 1,275 | (1,073, 1,477) | -1.2 |  |
| 3 | 35 | 2,740 | (2,333, 3,146) | 12.4 | 0.39 | 1,104 | (903, 1,305) | 10.2 | 0.48 | 1,476 | (1,204, 1,748) | 14.3 | 0.56 |
| **Saturated fat (% of total energy), tertiles** |  |  |  |  |  |  |  |  |  |  |  |  |  |
| 1 | 34 | 2,565 | (2,255, 2,874) | - |  | 1,047 | (892, 1,201) | - |  | 1,364 | (1,157, 1,572) | - |  |
| 2 | 35 | 2,617 | (2,323, 2,910) | 2.0 |  | 999 | (853, 1,146) | -4.6 |  | 1,476 | (1,279, 1,672) | 8.2 |  |
| 3 | 35 | 2,346 | (1,999, 2,694) | -8.5 | 0.56 | 1,003 | (829, 1,176) | -4.2 | 0.90 | 1,196 | (963, 1,429) | -12.3 | 0.26 |
| **Total fat (% of total energy), tertiles** |  |  |  |  |  |  |  |  |  |  |  |  |  |
| 1 | 34 | 2,513 | (2,219, 2,807) | - |  | 1,010 | (864, 1,156) | - |  | 1,359 | (1,162, 1,556) | - |  |
| 2 | 35 | 2,588 | (2,301, 2,876) | 3.0 |  | 1,000 | (857, 1,143) | -1.0 |  | 1,447 | (1,254, 1,640) | 6.5 |  |
| 3 | 35 | 2,428 | (2,115, 2,741) | -3.4 | 0.78 | 1,038 | (883, 1,193) | 2.8 | 0.94 | 1,232 | (1,022, 1,442) | -9.3 | 0.38 |
| **Total**^f^ **vitamin E/1,000 kcal, tertiles** |  |  |  |  |  |  |  |  |  |  |  |  |  |
| 1 | 34 | 2,277 | (1,944, 2,599) | - |  | 924 | (764, 1,084) | - |  | 1,222 | (1,002, 1,442) | - |  |
| 2 | 35 | 2,652 | (2,363, 2,941) | 16.5 |  | 1,096 | (952, 1,239) | 18.6 |  | 1,389 | (1,192, 1,587) | 13.7 |  |
| 3 | 35 | 2,598 | (2,296, 2,901) | 14.1 | 0.25 | 1,026 | (875, 1,176) | 11.0 | 0.33 | 1,428 | (1,222, 1,635) | 16.9 | 0.43 |
| **Total**^f^ **calcium/1,000 kcal, tertiles** |  |  |  |  |  |  |  |  |  |  |  |  |  |
| 1 | 34 | 2,595 | (2,284, 2,906) | - |  | 1,026 | (872, 1,180) | - |  | 1,423 | (1,214, 1,631) | - |  |
| 2 | 35 | 2,508 | (2,185, 2,830) | -3.4 |  | 972 | (812, 1,132) | -5.3 |  | 1,399 | (1,182, 1,615) | -1.7 |  |
| 3 | 35 | 2,431 | (2,114, 2,749) | -6.3 | 0.79 | 1,049 | (891, 1,206) | 2.2 | 0.81 | 1,223 | (1,010, 1,436) | -14.1 | 0.41 |
| **Dietary fiber/1,000 kcal, tertiles** |  |  |  |  |  |  |  |  |  |  |  |  |  |
| 1 | 34 | 2,355 | (1,978, 2,732) | - |  | 1,027 | (842, 1,213) | - |  | 1,163 | (911, 1,415) | - |  |
| 2 | 35 | 2,455 | (2,155, 2,754) | 4.2 |  | 1,012 | (865, 1,160) | -1.5 |  | 1,293 | (1,093, 1,494) | 11.2 |  |
| 3 | 35 | 2,720 | (2,328, 3,112) | 15.5 | 0.51 | 1,008 | (816, 1,201) | -1.9 | 0.99 | 1,581 | (1,319, 1,843) | 35.9 | 0.16 |
| **Total meat intake, tertiles** |  |  |  |  |  |  |  |  |  |  |  |  |  |
| 1 | 34 | 2,832 | (2,254, 3,210) | - |  | 1,124 | (937, 1,312) | - |  | 1,539 | (1,287, 1,791) | - |  |
| 2 | 35 | 2,411 | (2,109, 2,713) | -14.9 |  | 1,028 | (878, 1,178) | -8.5 |  | 1,228 | (1,027, 1,430) | -20.2 |  |
| 3 | 35 | 2,343 | (1,996, 2,689) | -17.3 | 0.18 | 917 | (744, 1,089) | -18.4 | 0.39 | 1,302 | (1,070, 1,533) | -15.4 | 0.17 |
| **Total vegetables and fruit intake, tertiles** |  |  |  |  |  |  |  |  |  |  |  |  |  |
| 1 | 34 | 2,761 | (2,418, 3,104) | - |  | 1,137 | (970, 1,305) | - |  | 1,464 | (1,229, 1,699) | - |  |
| 2 | 35 | 2,379 | (2,077, 2,681) | -13.8 |  | 931 | (784, 1,078) | -18.1 |  | 1,321 | (1,113, 1,528) | -9.8 |  |
| 3 | 35 | 2,402 | (2,050, 2,754) | -13.0 | 0.26 | 984 | (813, 1,156) | -13.5 | 0.22 | 1,260 | (1,019, 1,501) | -13.9 | 0.54 |
| **Serum 25-OH-vitamin D (ng/mL)** |  |  |  |  |  |  |  |  |  |  |  |  |  |
| < 17.9 | 34 | 2,552 | (2,245, 2,858) | - |  | 1,032 | (880, 1,183) | - |  | 1,363 | (1,156, 1,571) | - |  |
| 17.9 – 26.9 | 35 | 2,486 | (2,172, 2,800) | -2.6 |  | 964 | (803, 1,112) | -6.6 |  | 1,391 | (1,179, 1,603) | 2.1 |  |
| > 26.9 | 35 | 2,498 | (2,202, 2,794) | -2.1 | 0.70 | 1,052 | (913, 1,204) | 1.9 | 0.73 | 1,289 | (1,089, 1,489) | -5.4 | 0.79 |

Abbreviations: CI, confidence interval; NSAID, non-steroidal anti-inflammatory drug; OD, optical density; Prop. diff., proportional difference.

^a^ Biomarker expression measured using automated immunohistochemistry with image analysis. Adjusted mean differences in optical densities of biomarker labeling were calculated using general linear models, adjusted for staining batch, total energy intake, sex, and intakes of total meat, dietary fiber/1,000 kcal of total energy intake, and total vegetables and fruit.

^b^ Measured using automated immunohistochemistry with image analysis.

^c^ Calculated as (comparison group mean - reference group mean) / (reference group mean) x 100%.

^d^ Take at least once a week.

^e^ Low alcohol consumption is ≤ 0.8 drinks/day for men, 0 drinks/day for women; high alcohol consumption is > 0.8 drinks/day for men, > 0 drinks/day for women.

^f^ Dietary plus supplemental intake.

**Supplemental Table 2.** Comparisons^a^ of adjusted mean β-catenin expression^b^ in the normal-appearing colorectal mucosa of sporadic colorectal adenoma patients (*n* = 104), by selected participant characteristics.

| **Characteristics** | ***n*** | **Whole crypts, mean (OD)** | **95% CI** | **Prop. diff.**^c^ **(%)** | ***p*** | **Upper 40% of crypts, mean (OD)** | **95% CI** | **Prop. diff.**^c^ **(%)** | ***p*** | **Lower 60% of crypts, mean (OD)** | **95% CI** | **Prop. diff.**^c^ **(%)** | ***p*** |
| --- | --- | --- | --- | --- | --- | --- | --- | --- | --- | --- | --- | --- | --- |
| **Age (yrs)** |  |  |  |  |  |  |  |  |  |  |  |  |  |
| 47 – 54 | 34 | 10,255 | (9,178, 11,332) | - |  | 3,941 | (3,157, 4,725) | - |  | 6,227 | (5,578, 6,877) | - |  |
| 55 – 62 | 35 | 10,479 | (9,444, 11,514) | 2.18 |  | 3,814 | (3,060, 4,567) | -3.22 |  | 6,341 | (5,717, 6,965) | 1.83 |  |
| 63 – 75 | 35 | 10,807 | (9,737, 11,877) | 5.38 | 0.79 | 4,337 | (3,558, 5,116) | 10.05 | 0.65 | 6,407 | (5,762, 7,053) | 2.89 | 0.93 |
| **Sex** |  |  |  |  |  |  |  |  |  |  |  |  |  |
| Male | 48 | 9,369 | (7,151, 11,587) | - |  | 3,947 | (2,316, 5,577) | - |  | 5,361 | (4,036, 6,687) | - |  |
| Female | 56 | 11,510 | (9,588, 13,431) | 22.85 | 0.29 | 4,111 | (2,699, 5,524) | 4.16 | 0.91 | 7,156 | (6,008, 8,304) | 33.48 | 0.14 |
| **Regularly**^d^ **take aspirin** |  |  |  |  |  |  |  |  |  |  |  |  |  |
| No | 64 | 10,556 | (9,610, 11,502) | - |  | 4,031 | (3,340, 4,722) | - |  | 6,350 | (5,780, 6,919) | - |  |
| Yes | 40 | 10,500 | (9,773, 11,227) | -0.53 | 0.93 | 4,038 | (3,507, 4,569) | 0.17 | 0.99 | 6,314 | (5,877, 6,752) | -0.57 | 0.93 |
| **Regularly**^d^ **take other NSAID** |  |  |  |  |  |  |  |  |  |  |  |  |  |
| No | 69 | 10,696 | (9,679, 11,714) | - |  | 4,354 | (3,615, 5,092) | - |  | 6,427 | (5,814, 7,039) | - |  |
| Yes | 35 | 10,433 | (9,737, 11,129) | -2.46 | 0.69 | 3,874 | (3,369, 4,379) | -11.02 | 0.31 | 6,278 | (5,859, 6,696) | -2.32 | 0.70 |
| **Currently smoke** |  |  |  |  |  |  |  |  |  |  |  |  |  |
| No | 96 | 10,451 | (9,885, 11,017) | - |  | 4,003 | (3,588, 4,418) | - |  | 6,292 | (5,951, 6,634) | - |  |
| Yes | 8 | 11,368 | (9,236, 13,500) | 8.77 | 0.42 | 4,423 | (2,863, 5,983) | 10.49 | 0.61 | 6,755 | (5470, 8,039) | 7.36 | 0.50 |
| **Alcohol consumption** |  |  |  |  |  |  |  |  |  |  |  |  |  |
| Low^e^ | 57 | 10,447 | (9,587, 11,306) | - |  | 4,119 | (3,492, 4,746) | - |  | 6,258 | (5,741, 6,775) | - |  |
| High^e^ | 47 | 10,612 | (9,639, 11,586) | 1.58 | 0.82 | 3,933 | (3,223, 4,644) | -4.52 | 0.73 | 6,413 | (5,827, 6,998) | 2.48 | 0.73 |
| **Physical activity** |  |  |  |  |  |  |  |  |  |  |  |  |  |
| Low | 30 | 10,811 | (9,673, 11,947) | - |  | 3,910 | (3,084, 4,737) | - |  | 6,510 | (5,826, 7,193) | - |  |
| Moderate | 38 | 10,762 | (9,729, 11,795) | -0.45 |  | 3,729 | (2,977, 4,481) | -4.63 |  | 6,466 | (5,845, 7,088) | -0.68 |  |
| High | 27 | 10,187 | (9,273, 11,102) | -5.77 | 0.61 | 4,419 | (3,753, 5,085) | 13.02 | 0.36 | 6,124 | (5,574, 6,675) | -5.93 | 0.61 |
| **Body mass index (kg/m^2^)** |  |  |  |  |  |  |  |  |  |  |  |  |  |
| < 25.0 | 22 | 9,770 | (8,475, 11,064) | - |  | 4,446 | (3,498, 5,394) | - |  | 5,867 | (5,088, 6,645) | - |  |
| 25.0 – 29.9 | 43 | 10,820 | (9,944, 11,696) | 10.75 |  | 3,801 | (3,159, 4,442) | -14.51 |  | 6,520 | (5,994, 7,047) | 11.13 |  |
| ≥ 30 | 39 | 10,616 | (9,653, 11,579) | 8.66 | 0.41 | 4,063 | (3,357, 4,768) | -8.61 | 0.53 | 6,375 | (5,796, 6,955) | 8.66 | 0.39 |
| **Total energy, tertiles** |  |  |  |  |  |  |  |  |  |  |  |  |  |
| 1 | 34 | 9,781 | (8,761, 10,802) | - |  | 4,431 | (3,671, 5,190) | - |  | 5,920 | (5,303, 6,536) | - |  |
| 2 | 35 | 11,380 | (10,381, 12,379) | 16.35 |  | 4,052 | (3,310, 4,795) | -8.55 |  | 6,809 | (6,206, 7,412) | 15.02 |  |
| 3 | 35 | 10,336 | (9,341, 11,331) | 5.67 | 0.10 | 3,645 | (2,905, 4,385) | -17.74 | 0.36 | 6,218 | (5,617, 6,819) | 5.03 | 0.14 |
| **Saturated fat (% of total energy), tertiles** |  |  |  |  |  |  |  |  |  |  |  |  |  |
| 1 | 34 | 10,607 | (9,256, 11,958) | - |  | 3,629 | (2,652, 4,607) | - |  | 6,420 | (5,608, 7,232) | - |  |
| 2 | 35 | 11,158 | (10,176, 12,139) | 5.19 |  | 4,624 | (3,914, 5,334) | 27.42 |  | 6,714 | (6,124, 7,304) | 4.58 |  |
| 3 | 35 | 9,762 | (8,316, 11,209) | -7.97 | 0.27 | 3,818 | (2,772, 4,865) | 5.21 | 0.14 | 5,827 | (4,958, 6,697) | -9.24 | 0.25 |
| **Total fat (% of total energy), tertiles** |  |  |  |  |  |  |  |  |  |  |  |  |  |
| 1 | 34 | 11,213 | (10,201, 12,224) | - |  | 3,945 | (3,205, 4,684) | - |  | 6,729 | (6,120, 7,339) | - |  |
| 2 | 35 | 10,534 | (9,570, 11,498) | -6.06 |  | 4,249 | (3,544, 4,954) | 7.71 |  | 6,372 | (5,792, 6,953) | -5.31 |  |
| 3 | 35 | 9,817 | (8,798, 10,836) | -12.45 | 0.19 | 3,900 | (3,155, 4,645) | -1.14 | 0.30 | 5,879 | (5,265, 6,493) | -12.63 | 0.18 |
| **Total**^f^ **vitamin E/1,000 kcal, tertiles** |  |  |  |  |  |  |  |  |  |  |  |  |  |
| 1 | 34 | 11,096 | (9,955, 12,236) | - |  | 3,804 | (2,980, 4,629) | - |  | 6,691 | (6,005, 7,376) | - |  |
| 2 | 35 | 10,344 | (9,333, 11,355) | -6.78 |  | 3,677 | (2,946, 4,408) | -3.34 |  | 6,170 | (5,562, 6,777) | -7.79 |  |
| 3 | 35 | 10,141 | (9,003, 11,279) | -8.61 | 0.53 | 4,618 | (3,795, 5,441) | 21.40 | 0.25 | 6,133 | (5,449, 6,818) | -8.34 | 0.49 |
| **Total**^f^ **calcium/1,000 kcal, tertiles** |  |  |  |  |  |  |  |  |  |  |  |  |  |
| 1 | 34 | 10,141 | (9,054, 11,229) | - |  | 3,785 | (2,999, 4,571) | - |  | 6,090 | (5,435, 6,745) | - |  |
| 2 | 35 | 10,511 | (9,443, 11,578) | 3.65 |  | 4,554 | (3,782, 5,325) | 20.32 |  | 6,375 | (5,732, 7,018) | 4.68 |  |
| 3 | 35 | 10,912 | (9,849, 11,975) | 7.60 | 0.64 | 3,782 | (3,013, 4,550) | -0.08 | 0.30 | 6,520 | (5,880, 7,160) | 7.06 | 0.68 |
| **Dietary fiber/1,000 kcal, tertiles** |  |  |  |  |  |  |  |  |  |  |  |  |  |
| 1 | 34 | 10,899 | (9,647, 12,151) | - |  | 3,610 | (2,706, 4,515) | - |  | 6,544 | (5,790, 7,298) | - |  |
| 2 | 35 | 10,302 | (9,211, 11,394) | -5.48 |  | 4,493 | (3,704, 5,281) | 24.46 |  | 6,207 | (5,550, 6,864) | -5.15 |  |
| 3 | 35 | 10,374 | (9,070, 11,677) | -4.82 | 0.78 | 3,991 | (3,050, 4,933) | 10.55 | 0.34 | 6,239 | (5,454, 7,024) | -4.66 | 0.81 |
| **Total meat intake, tertiles** |  |  |  |  |  |  |  |  |  |  |  |  |  |
| 1 | 34 | 9,198 | (7,910, 10,487) | - |  | 4,288 | (3,322, 5,254) | - |  | 5,537 | (4,763, 6,311) | - |  |
| 2 | 35 | 11,086 | (10,100, 12,073) | 20.53 |  | 3,690 | (2,950, 4,429) | -13.95 |  | 6,707 | (6,114, 7,300) | 21.13 |  |
| 3 | 35 | 11,081 | (9,909, 12,253) | 20.47 | 0.07 | 4,147 | (3,269, 5,026) | -3.29 | 0.53 | 6,623 | (5,919, 7,328) | 19.61 | 0.06 |
| **Total vegetables and fruit intake, tertiles** |  |  |  |  |  |  |  |  |  |  |  |  |  |
| 1 | 34 | 10,704 | (9,548, 11,860) | - |  | 3,732 | (2,894, 4,570) | - |  | 6,381 | (5,685, 7,076) | - |  |
| 2 | 35 | 10,382 | (9,342, 11,423) | -3.01 |  | 4,382 | (3,628, 5,136) | 17.42 |  | 6,225 | (5,599, 6,852) | -2.44 |  |
| 3 | 35 | 10,486 | (9,360, 11,612) | -2.04 | 0.93 | 3,972 | (3,156, 4,788) | 6.43 | 0.54 | 6,383 | (5,706, 7,061) | 0.03 | 0.93 |
| **Serum 25-OH-vitamin D (ng/mL)** |  |  |  |  |  |  |  |  |  |  |  |  |  |
| < 17.9 | 34 | 11,267 | (10,259, 12,275) | - |  | 3,926 | (3,170, 4,683) | - |  | 6,817 | (6,212, 7,421) | - |  |
| 17.9 – 26.9 | 35 | 9,540 | (8,521, 10,559) | -15.33 |  | 4,367 | (3,602, 5,133) | 11.23 |  | 5,722 | (5,110, 6,333) | -16.06 |  |
| > 26.9 | 35 | 10,779 | (9,767, 11,790) | -4.33 | 0.07 | 3,809 | (3,050, 4,569) | -2.98 | 0.59 | 6,459 | (5,852, 7,066) | -5.25 | 0.05 |

Abbreviations: CI, confidence interval; NSAID, non-steroidal anti-inflammatory drug; OD, optical density; Prop. diff., proportional difference.

^a^ Biomarker expression measured using automated immunohistochemistry with image analysis. Adjusted mean differences in optical densities of biomarker labeling were calculated using general linear models, adjusted for staining batch, total energy intake, and total fat intake as a percentage of total energy intake.

^b^ Measured using automated immunohistochemistry with image analysis.

^c^ Calculated as (comparison group mean - reference group mean) / (reference group mean) x 100%.

^d^ Take at least once a week.

^e^ Low alcohol consumption is ≤ 0.8 drinks/day for men, 0 drinks/day for women; high alcohol consumption is > 0.8 drinks/day for men, > 0 drinks/day for women.

^f^ Dietary plus supplemental intake.

**Supplemental Table 3.** Comparisons^a^ of adjusted mean APC/β-catenin expression^b^ and MSH2/mib-1 expression^c^ in the normal-appearing colorectal mucosa of sporadic colorectal adenoma patients (*n* = 104), by selected participant characteristics.

|  | **APC/β-catenin**^b^ | | | | | | | | | | | | | | | | | | | | | | | | | | | **MSH2/mib-1**^c^ | | | | | | | | | |  |
| --- | --- | --- | --- | --- | --- | --- | --- | --- | --- | --- | --- | --- | --- | --- | --- | --- | --- | --- | --- | --- | --- | --- | --- | --- | --- | --- | --- | --- | --- | --- | --- | --- | --- | --- | --- | --- | --- | --- |
| **Characteristics** | | ***n*** | | **Whole crypts, mean (OD)** | | **95% CI** | | **Prop. diff.**^d^ **(%)** | | ***p*** | | **Upper 40% of crypts, mean (OD)** | | **95% CI** | | **Prop. diff.^d^ (%)** | | ***p*** | | **Lower 60% of crypts, mean (OD)** | | **95% CI** | | **Prop. diff.**^d^**(%)** | | ***p*** | | | **Whole crypts, mean (OD)** | | **95% CI** | | **Prop. diff.**^d^ **(%)** | | ***p*** | |  |  |
| **Age (yrs)** | | |  | |  | |  | |  | |  | |  | |  | |  | |  | |  | |  | |  | |  | | |  | |  | |  | |  | | |
| 47 – 54 | | 34 | | 0.23 | | (0.19, 0.27) | | - | |  | | 0.20 | | (0.16, 0.24) | | - | |  | | 0.26 | | (0.20, 0.32) | | - | |  | | | 1.62 | | (1.38, 1.85) | | - | |  | |  |  |
| 55 – 62 | | 35 | | 0.25 | | (0.21, 0.29) | | 8.7 | |  | | 0.22 | | (0.18, 0.26) | | 10.0 | |  | | 0.29 | | (0.23, 0.34) | | 11.5 | |  | | | 1.59 | | (1.37, 1.82) | | -1.9 | |  | |  |  |
| 63 – 75 | | 35 | | 0.23 | | (0.18, 0.27) | | 0.0 | | 0.68 | | 0.20 | | (0.16, 0.24) | | 0.0 | | 0.69 | | 0.25 | | (0.20, 0.31) | | -3.8 | | 0.75 | | | 1.62 | | (1.39, 1.86) | | 0.0 | | 0.98 | |  |  |
| **Sex** | |  | |  | |  | |  | |  | |  | |  | |  | |  | |  | |  | |  | |  | | |  | |  | |  | |  | |  |  |
| Male | | 48 | | 0.12 | | (0.02, 0.21) | | - | |  | | 0.10 | | (0.01, 0.20) | | - | |  | | 0.13 | | (-0.003, 0.27) | | - | |  | | | 1.83 | | (1.46, 2.21) | | - | |  | |  |  |
| Female | | 56 | | 0.35 | | (0.27, 0.43) | | 191.7 | | 0.01 | | 0.31 | | (0.23, 0.39) | | 210.0 | | 0.02 | | 0.40 | | (0.29, 0.52) | | 207.7 | | 0.03 | | | 1.43 | | (1.11, 1.74) | | -21.9 | | 0.22 | |  |  |
| **Regularly**^e^ **take aspirin** | |  | |  | |  | |  | |  | |  | |  | |  | |  | |  | |  | |  | |  | | |  | |  | |  | |  | |  |  |
| No | | 64 | | 0.24 | | (0.20, 0.27) | | - | |  | | 0.20 | | (0.16, 0.23) | | - | |  | | 0.29 | | (0.24, 0.34) | | - | |  | | | 1.75 | | (1.55, 1.94) | | - | |  | |  |  |
| Yes | | 40 | | 0.23 | | (0.20, 0.26) | | -4.2 | | 0.84 | | 0.21 | | (0.18, 0.24) | | 5.0 | | 0.57 | | 0.25 | | (0.21, 0.30) | | -13.8 | | 0.34 | | | 1.48 | | (1.32, 1.63) | | -15.4 | | 0.04 | |  |  |
| **Regularly**^e^ **take other NSAID** | |  | |  | |  | |  | |  | |  | |  | |  | |  | |  | |  | |  | |  | | |  | |  | |  | |  | |  |  |
| No | | 69 | | 0.23 | | (0.19, 0.27) | | - | |  | | 0.21 | | 0.18, 0.25) | | - | |  | | 0.24 | | (0.19, 0.30) | | - | |  | | | 1.63 | | (1.42, 1.85) | | - | |  | |  |  |
| Yes | | 35 | | 0.24 | | (0.21, 0.26) | | 4.3 | | 0.76 | | 0.20 | | (0.18, 0.23) | | -4.8 | | 0.61 | | 0.28 | | (0.24, 0.32) | | 16.7 | | 0.30 | | | 1.60 | | (1.46, 1.75) | | -1.8 | | 0.82 | |  |  |
| **Currently smoke** | |  | |  | |  | |  | |  | |  | |  | |  | |  | |  | |  | |  | |  | | |  | |  | |  | |  | |  |  |
| No | | 96 | | 0.24 | | (0.21, 0.26) | | - | |  | | 0.21 | | (0.18, 0.23) | | - | |  | | 0.27 | | (0.24, 0.30) | | - | |  | | | 1.62 | | (1.50, 1.74) | | - | |  | |  |  |
| Yes | | 8 | | 0.21 | | (0.13, 0.30) | | -12.5 | | 0.58 | | 0.20 | | (0.11, 0.28) | | -4.8 | | 0.78 | | 0.23 | | (0.12, 0.35) | | -14.8 | | 0.56 | | | 1.43 | | (0.97, 1.89) | | -11.7 | | 0.43 | |  |  |
| **Alcohol consumption** | |  | |  | |  | |  | |  | |  | |  | |  | |  | |  | |  | |  | |  | | |  | |  | |  | |  | |  |  |
| Low^f^ | | 57 | | 0.26 | | (0.23, 0.29) | | - | |  | | 0.23 | | (0.19, 0.26) | | - | |  | | 0.29 | | (0.25, 0.34) | | - | |  | | | 1.69 | | (1.51, 1.87) | | - | |  | |  |  |
| High^f^ | | 47 | | 0.23 | | (0.19, 0.27) | | -11.5 | | 0.30 | | 0.20 | | (0.17, 0.24) | | -13.0 | | 0.39 | | 0.26 | | (0.21, 0.31) | | -10.3 | | 0.42 | | | 1.44 | | (1.24, 1.65) | | -14.8 | | 0.12 | |  |  |
| **Physical activity** | |  | |  | |  | |  | |  | |  | |  | |  | |  | |  | |  | |  | |  | | |  | |  | |  | |  | |  |  |
| Low | | 30 | | 0.23 | | (0.19, 0.27) | | - | |  | | 0.20 | | (0.16, 0.24) | | - | |  | | 0.27 | | (0.21, 0.33) | | - | |  | | | 1.70 | | (1.46, 1.93) | | - | |  | |  |  |
| Moderate | | 38 | | 0.24 | | (0.19, 0.28) | | 4.3 | |  | | 0.21 | | (0.17, 0.25) | | 5.0 | |  | | 0.27 | | (0.21, 0.32) | | 0.0 | |  | | | 1.52 | | (1.31, 1.74) | | -10.6 | |  | |  |  |
| High | | 27 | | 0.23 | | (0.19, 0.27) | | 0.0 | | 0.99 | | 0.21 | | (0.17, 0.24) | | 5.0 | | 0.93 | | 0.26 | | (0.21, 0.32) | | -3.7 | | 0.99 | | | 1.62 | | (1.43, 1.82) | | -4.7 | | 0.55 | |  |  |
| **Body mass index (kg/m^2^)** | |  | |  | |  | |  | |  | |  | |  | |  | |  | |  | |  | |  | |  | | |  | |  | |  | |  | |  |  |
| < 25.0 | | 22 | | 0.22 | | (0.17, 0.28) | | - | |  | | 0.20 | | (0.15, 0.26) | | - | |  | | 0.25 | | (0.18, 0.33) | | - | |  | | | 1.61 | | (1.33, 1.89) | | - | |  | |  |  |
| 25.0 – 29.9 | | 43 | | 0.22 | | (0.19, 0.26) | | 0.0 | |  | | 0.20 | | (0.16, 0.23) | | 0.0 | |  | | 0.26 | | (0.21, 0.31) | | 4.0 | |  | | | 1.64 | | (1.45, 1.83) | | 1.9 | |  | |  |  |
| ≥ 30 | | 39 | | 0.25 | | (0.21, 0.29) | | 13.6 | | 0.62 | | 0.22 | | (0.18, 0.26) | | 10.0 | | 0.69 | | 0.29 | | (0.23, 0.34) | | 16.0 | | 0.73 | | | 1.58 | | (1.38, 1.79) | | -1.9 | | 0.94 | |  |  |
| **Total energy, tertiles** | |  | |  | |  | |  | |  | |  | |  | |  | |  | |  | |  | |  | |  | | |  | |  | |  | |  | |  |  |
| 1 | | 34 | | 0.24 | | (0.19, 0.29) | | - | |  | | 0.20 | | (0. 16, 0.25) | | - | |  | | 0.29 | | (0.22, 0.36) | | - | |  | | | 1.71 | | (1.49, 1.93) | | - | |  | |  |  |
| 2 | | 35 | | 0.20 | | (0.16, 0.24) | | -16.7 | |  | | 0.18 | | (0.14, 0.22) | | -10.0 | |  | | 0.22 | | (0.16, 0.27) | | -24.1 | |  | | | 1.44 | | (1.23, 1.65) | | -15.8 | |  | |  |  |
| 3 | | 35 | | 0.27 | | (0.22, 0.32) | | 12.5 | | 0.09 | | 0.24 | | (0.19, 0.29) | | 20.0 | | 0.19 | | 0.30 | | (0.23, 0.38) | | 3.4 | | 0.11 | | | 1.69 | | (1.48, 1.90) | | -1.2 | | 0.18 | |  |  |
| Saturated fat (% of total energy), tertiles | |  | |  | |  | |  | |  | |  | |  | |  | |  | |  | |  | |  | |  | | |  | |  | |  | |  | |  |  |
| 1 | | 34 | | 0.24 | | (0.18, 0.29) | | - | |  | | 0.20 | | (0.15, 0.25) | | - | |  | | 0.29 | | (0.21, 0.37) | | - | |  | | | 1.46 | | (1.23, 1.68) | | - | |  | |  |  |
| 2 | | 35 | | 0.22 | | (0.18, 0.26) | | -8.3 | |  | | 0.21 | | (0.17, 0.25) | | 5.0 | |  | | 0.24 | | (0.18, 0.29) | | -17.2 | |  | | | 1.70 | | (1.48, 1.91) | | 16.4 | |  | |  |  |
| 3 | | 35 | | 0.24 | | (0.19, 0.30) | | 0.0 | | 0.79 | | 0.21 | | (0.16, 0.27) | | 5.0 | | 0.96 | | 0.28 | | (0.20, 0.36) | | -3.4 | | 0.36 | | | 1.68 | | (1.44, 1.91) | | 15.1 | | 0.25 | |  |  |
| Total fat (% of total energy), tertiles | |  | |  | |  | |  | |  | |  | |  | |  | |  | |  | |  | |  | |  | | |  | |  | |  | |  | |  |  |
| 1 | | 34 | | 0.21 | | (0.14, 0.28) | | - | |  | | 0.20 | | (0.16, 0.24) | | - | |  | | 0.24 | | (0.12, 0.31) | | - | |  | | | 1.53 | | (1.31, 1.75) | | - | |  | |  |  |
| 2 | | 35 | | 0.24 | | (0.20, 0.28) | | 14.3 | |  | | 0.22 | | (0. 18, 0.26) | | 10.0 | |  | | 0.27 | | (0.21, 0.33) | | 12.5 | |  | | | 1.64 | | (1.42, 1.86) | | 7.2 | |  | |  |  |
| 3 | | 35 | | 0.25 | | (0.18, 0.32) | | 19.0 | | 0.75 | | 0.20 | | (0.16, 0.24) | | 0.0 | | 0.78 | | 0.29 | | (0.22, 0.41) | | 20.8 | | 0.49 | | | 1.66 | | (1.44, 1.88) | | 8.5 | | 0.66 | |  |  |
| Totalg vitamin E/1,000 kcal, tertiles | |  | |  | |  | |  | |  | |  | |  | |  | |  | |  | |  | |  | |  | | |  | |  | |  | |  | |  |  |
| 1 | | 34 | | 0.21 | | (0.15, 0.26) | | - | |  | | 0.18 | | (0.12, 0.23) | | - | |  | | 0.24 | | (0.17, 0.32) | | - | |  | | | 1.65 | | (1.42, 1.88) | | - | |  | |  |  |
| 2 | | 35 | | 0.25 | | (0.21, 0.29) | | 19.0 | |  | | 0.21 | | (0.17, 0.25) | | 16.7 | |  | | 0.30 | | (0.25, 0.36) | | 25.0 | |  | | | 1.55 | | (1.34, 1.77) | | -6.1 | |  | |  |  |
| 3 | | 35 | | 0.24 | | (0.19, 0.29) | | 14.3 | | 0.39 | | 0.23 | | (0.17, 0.28) | | 27.8 | | 0.41 | | 0.25 | | (0.18, 0.33) | | 4.2 | | 0.35 | | | 1.63 | | (1.40, 1.86) | | -1.2 | | 0.81 | |  |  |
| Totalg calcium/1,000 kcal, tertiles | |  | |  | |  | |  | |  | |  | |  | |  | |  | |  | |  | |  | |  | | |  | |  | |  | |  | |  |  |
| 1 | | 34 | | 0.25 | | (0.21, 0.29) | | - | |  | | 0.23 | | (0.18, 0.25) | | - | |  | | 0.27 | | (0.21, 0.33) | | - | |  | | | 1.67 | | (1.45, 1.90) | | - | |  | |  |  |
| 2 | | 35 | | 0.23 | | (0.18, 0.27) | | -8.0 | |  | | 0.20 | | (0.16, 0.25) | | -13.0 | |  | | 0.27 | | (0.20, 0.33) | | 0.0 | |  | | | 1.56 | | (1.30, 1.81) | | -6.6 | |  | |  |  |
| 3 | | 35 | | 0.23 | | (0.18, 0.27) | | -8.0 | | 0.73 | | 0.19 | | (0.15, 0.23) | | -17.4 | | 0.53 | | 0.26 | | (0.20, 0.33) | | -3.7 | | 0.98 | | | 1.60 | | (1.36, 1.83) | | -4.2 | | 0.80 | |  |  |
| Dietary fiber/1,000 kcal, tertiles | |  | |  | |  | |  | |  | |  | |  | |  | |  | |  | |  | |  | |  | | |  | |  | |  | |  | |  |  |
| 1 | | 34 | | 0.25 | | (0.18, 0.31) | | - | |  | | 0.20 | | (0.13, 0.26) | | - | |  | | 0.25 | | (0.21, 0.40) | | - | |  | | | 1.76 | | (1.51, 2.00) | | - | |  | |  |  |
| 2 | | 35 | | 0.24 | | (0.20, 0.29) | | -4.0 | |  | | 0.21 | | (0.16, 0.25) | | 5.0 | |  | | 0.28 | | (0.23, 0.35) | | 12.0 | |  | | | 1.65 | | (1.43, 1.87) | | -6.3 | |  | |  |  |
| 3 | | 35 | | 0.21 | | (0.14, 0.29) | | -16.0 | | 0.82 | | 0.21 | | (0.14, 0.29) | | 5.0 | | 0.96 | | 0.26 | | (0.10, 0.31) | | 4.0 | | 0.78 | | | 1.42 | | (1.17, 1.67) | | -19.3 | | 0.24 | |  |  |
| Total meat intake, tertiles | |  | |  | |  | |  | |  | |  | |  | |  | |  | |  | |  | |  | |  | | |  | |  | |  | |  | |  |  |
| 1 | | 34 | | 0.29 | | (0.23, 0.35) | | - | |  | | 0.25 | | (0.19, 0.31) | | - | |  | | 0.34 | | (0.27, 0.42) | | - | |  | | | 1.52 | | (1.24, 1.80) | | - | |  | |  |  |
| 2 | | 35 | | 0.21 | | (0.17, 0.26) | | -27.6 | |  | | 0.18 | | (0.14, 0.22) | | -28.0 | |  | | 0.25 | | (0.19, 0.31) | | -26.5 | |  | | | 1.69 | | (1.46, 1.91) | | 11.2 | |  | |  |  |
| 3 | | 35 | | 0.21 | | (0.15, 0.26) | | -27.6 | | 0.11 | | 0.20 | | (0.14, 0.25) | | -20.0 | | 0.15 | | 0.22 | | (0.16, 0.28) | | -35.3 | | 0.08 | | | 1.61 | | (1.36, 1.87) | | 5.9 | | 0.63 | |  |  |
| Total vegetables and fruit intake, tertiles | |  | |  | |  | |  | |  | |  | |  | |  | |  | |  | |  | |  | |  | | |  | |  | |  | |  | |  |  |
| 1 | | 34 | | 0.25 | | (0.19, 0.32) | | - | |  | | 0.22 | | (0.16, 0.28) | | - | |  | | 0.28 | | (0.21, 0.35) | | - | |  | | | 1.72 | | (1.48, 1.96) | | - | |  | |  |  |
| 2 | | 35 | | 0.23 | | (0.18, 0.27) | | -8.0 | |  | | 0.20 | | (0.16, 0.24) | | -9.1 | |  | | 0.26 | | (0.20, 0.32) | | -7.1 | |  | | | 1.65 | | (1.43, 1.86) | | -4.1 | |  | |  |  |
| 3 | | 35 | | 0.22 | | (0.15, 0.29) | | -12.0 | | 0.76 | | 0.20 | | (0.13, 0.27) | | -9.1 | | 0.88 | | 0.26 | | (0.19, 0.33) | | -7.1 | | 0.87 | | | 1.46 | | (1.24, 1.69) | | -15.1 | | 0.32 | |  |  |
| Serum 25-OH-vitamin D (ng/mL) | |  | |  | |  | |  | |  | |  | |  | |  | |  | |  | |  | |  | |  | | |  | |  | |  | |  | |  |  |
| < 17.9 | | 34 | | 0.23 | | (0.19, 0.27) | | - | |  | | 0.19 | | (0.15, 0.24) | | - | |  | | 0.27 | | (0.21, 0.33) | | - | |  | | | 1.49 | | (1.27, 1.71) | | - | |  | |  |  |
| 17.9 – 26.9 | | 35 | | 0.25 | | (0.20, 0.29) | | 8.7 | |  | | 0.23 | | (0.18, 0.27) | | 21.1 | |  | | 0.27 | | (0.21, 0.33) | | 0.0 | |  | | | 1.75 | | (1.52, 1.98) | | 17.4 | |  | |  |  |
| > 26.9 | | 35 | | 0.22 | | (0.18, 0.27) | | -4.3 | | 0.74 | | 0.19 | | (0.15, 0.23) | | 0.0 | | 0.45 | | 0.26 | | (0.20, 0.32) | | -3.7 | | 0.97 | | | 1.59 | | (1.37, 1.82) | | 6.7 | | 0.30 | |  |  |

Abbreviations: CI, confidence interval; NSAID, non-steroidal anti-inflammatory drug; OD, optical density; Prop. diff., proportional difference.

^a^ Biomarker expression measured using automated immunohistochemistry with image analysis. Adjusted mean differences in optical densities of biomarker labeling were calculated using general linear models, adjusted for staining batch, total energy intake, and other confounding variables (for APC/β-catenin models: sex and intakes of total meat, dietary fiber/1,000 kcal of total energy intake, and total vegetables and fruit; for MSH2/mib-1 models: aspirin use and total calcium intake/1,000 kcal of total energy intake).

^b^ APC expression divided by β-catenin expression.

^c^ MSH2 expression divided by mib-1 expression.

^d^ Calculated as (comparison group mean - reference group mean) / (reference group mean) x 100%.

^e^ Take at least once a week.

^f^ Low alcohol consumption is ≤ 0.8 drinks/day for men, 0 drinks/day for women; high alcohol consumption is > 0.8 drinks/day for men, > 0 drinks/day for women.

^g^ Dietary plus supplemental intake.

**Supplemental Table 4.** Comparisons^a^ of adjusted mean E-cadherin expression^b^ in the normal-appearing colorectal mucosa of sporadic colorectal adenoma patients (n = 104), by selected participant characteristics.

| **Characteristics** | ***n*** | **Whole crypts, mean (OD)** | **95% CI** | **Prop. diff.**^c^ **(%)** | ***p*** | **Upper 40% of crypts, mean (OD)** | **95% CI** | **Prop. diff.**^c^ **(%)** | ***p*** | **Lower 60% of crypts, mean (OD)** | **95% CI** | **Prop. diff.**^c^ **(%)** | ***p*** |
| --- | --- | --- | --- | --- | --- | --- | --- | --- | --- | --- | --- | --- | --- |
| **Age (yrs)** |  |  |  |  |  |  |  |  |  |  |  |  |  |
| 47 – 54 | 34 | 4,611 | (4,026, 5,196) | - |  | 3,083 | (1,975, 4,191) | - |  | 2,735 | (2,373, 3,097) | - |  |
| 55 – 62 | 35 | 4,741 | (4,175, 5,306) | 2.8 |  | 1,957 | (886, 3,028) | -36.5 |  | 2,836 | (2,486, 3,186) | 3.7 |  |
| 63 – 75 | 35 | 4,416 | (3,845, 4,986) | -4.2 | 0.75 | 1,987 | (907, 3,066) | -35.5 | 0.29 | 2,580 | (2,228, 2,933) | -5.7 | 0.63 |
| **Sex** |  |  |  |  |  |  |  |  |  |  |  |  |  |
| Male | 48 | 4,423 | (3,481, 5,365) | - |  | 1,377 | (-417, 3,170) | - |  | 2,586 | (2,002, 3,170) | - |  |
| Female | 56 | 4,728 | (3,907, 5,549) | 6.9 | 0.72 | 3,137 | (1,574, 4,701) | 127.8 | 0.27 | 2,827 | (2,317, 3,336) | 9.3 | 0.64 |
| **Regularly**^d^ **take aspirin** |  |  |  |  |  |  |  |  |  |  |  |  |  |
| No | 64 | 4,648 | (4,132, 5,163) | - |  | 2,385 | (1,396, 3,374) | - |  | 2,763 | (2,443, 3,082) | - |  |
| Yes | 40 | 4,549 | (4,155, 4,943) | -2.1 | 0.78 | 2,287 | (1,531, 3,043) | -4.1 | 0.88 | 2,686 | (2,441, 2,930) | -2.8 | 0.72 |
| **Regularly**^d^ **take other NSAID** |  |  |  |  |  |  |  |  |  |  |  |  |  |
| No | 69 | 4,609 | (4,061, 5,158) | - |  | 1,872 | (826, 2,917) | - |  | 2,722 | (2,381, 3,062) | - |  |
| Yes | 35 | 4,576 | (4,201, 4,950) | -0.7 | 0.92 | 2,554 | (1,841, 3,268) | 36.4 | 0.31 | 2,712 | (2,480, 2,945) | -0.4 | 0.97 |
| **Currently smoke** |  |  |  |  |  |  |  |  |  |  |  |  |  |
| No | 96 | 4,615 | (4,310, 4,920) | - |  | 2,233 | (1,650, 2,816) | - |  | 2,736 | (2,547, 2,925) | - |  |
| Yes | 8 | 4,250 | (3,080, 5,421) | -7.9 | 0.56 | 3,425 | (1,191, 5,659) | 53.4 | 0.31 | 2,470 | (1,745, 3,196) | -9.7 | 0.49 |
| **Alcohol consumption** |  |  |  |  |  |  |  |  |  |  |  |  |  |
| Low^e^ | 57 | 4,669 | (4,221, 5,117) |  |  | 2,359 | (1,499, 3,219) |  |  | 2,759 | (2,481, 3,037) |  |  |
| High^e^ | 47 | 4,487 | (3,982, 4,992) | -3.9 | 0.63 | 2,283 | (1,313, 3,254) | -3.2 | 0.92 | 2,662 | (2,349, 2,976) | -3.5 | 0.68 |
| **Physical activity** |  |  |  |  |  |  |  |  |  |  |  |  |  |
| Low | 30 | 4,829 | (4,234, 5,423) | - |  | 2,429 | (1,322, 3,537) | - |  | 2,897 | (2,529, 3,265) | - |  |
| Moderate | 38 | 4,728 | (4,193, 5,264) | -2.1 |  | 3,463 | (2,466, 4,460) | 42.6 |  | 2,789 | (2,457, 3,120) | -3.7 |  |
| High | 27 | 4,342 | (3,841, 4,842) | -10.1 | 0.43 | 1,345 | (413, 2,277) | -44.6 | 0.01 | 2,546 | (2,237, 2,856) | -12.1 | 0.36 |
| **Body mass index (kg/m^2^)** |  |  |  |  |  |  |  |  |  |  |  |  |  |
| < 25.0 | 22 | 4,471 | (3,770, 5,173) | - |  | 1,143 | (-169, 2,456) | - |  | 2,629 | (2,194, 3,064) | - |  |
| 25.0 – 29.9 | 43 | 4,580 | (4,084, 5,075) | 2.4 |  | 2,674 | (1,746, 3,601) | 133.9 |  | 2,708 | (2,400, 3,015) | 3.0 |  |
| ≥ 30 | 39 | 4,661 | (4,140, 5,181) | 4.2 | 0.92 | 2,606 | (1,633, 3,579) | 128.0 | 0.15 | 2,773 | (2,450, 3,095) | 5.5 | 0.87 |
| **Total energy, tertiles** |  |  |  |  |  |  |  |  |  |  |  |  |  |
| 1 | 34 | 4,659 | (4,086, 5,232) | - |  | 1,747 | (644, 2,850) | - |  | 2,810 | (2,454, 3,166) | - |  |
| 2 | 35 | 4,232 | (3,695, 4,768) | -9.2 |  | 2,647 | (1,614, 3,679) | 51.5 |  | 2,528 | (2,195, 2,861) | -10.0 |  |
| 3 | 35 | 4,884 | (4,333, 5,436) | 4.8 | 0.26 | 2,538 | (1,476, 3,600) | 45.3 | 0.73 | 2,819 | (2,476, 3,161) | 0.3 | 0.42 |
| **Saturated fat (% of total energy), tertiles** |  |  |  |  |  |  |  |  |  |  |  |  |  |
| 1 | 34 | 4,284 | (3,725, 4,843) | - |  | 2,160 | (1,060, 3,259) | - |  | 2,519 | (2,172, 2,866) | - |  |
| 2 | 35 | 5,046 | (4,504, 5,589) | 17.8 |  | 2,645 | (1,578, 3,712) | 22.5 |  | 2,991 | (2,655, 3,328) | 18.7 |  |
| 3 | 35 | 4,403 | (3,764, 5,043) | 2.8 | 0.12 | 2,151 | (892, 3,409) | -0.4 | 0.77 | 2,620 | (2,222, 3,017) | 4.0 | 0.13 |
| **Total fat (% of total energy), tertiles** |  |  |  |  |  |  |  |  |  |  |  |  |  |
| 1 | 34 | 4,435 | (3,888, 4,982) | - |  | 2,392 | (1,334, 3,450) | - |  | 2,589 | (2,250, 2,928) | - |  |
| 2 | 35 | 4,885 | (4,340, 5,430) | 10.1 |  | 2,085 | (1,031, 3,139) | -12.8 |  | 2,913 | (2,576, 3,250) | 12.5 |  |
| 3 | 35 | 4,424 | (3,861, 4,987) | -0.2 | 0.44 | 2,512 | (1,244, 3,601) | 5.0 | 0.86 | 2,633 | (2,284, 2,981) | 1.7 | 0.38 |
| **Total**^f^ **vitamin E/1,000 kcal, tertiles** |  |  |  |  |  |  |  |  |  |  |  |  |  |
| 1 | 34 | 4,769 | (4,152, 5,385) | - |  | 2,413 | (1,240, 3,587) | - |  | 2,878 | (2,497, 3,259) | - |  |
| 2 | 35 | 4,507 | (3,953, 5,060) | -5.5 |  | 2,749 | (1,694, 3,803) | 13.9 |  | 2,625 | (2,283, 2,968) | -8.8 |  |
| 3 | 35 | 4,491 | (3,908, 5,074) | -5.8 | 0.80 | 1,815 | (704, 2,926) | -24.8 | 0.49 | 2,648 | (2,287, 3,008) | -8.0 | 0.62 |
| **Total**^f^ **calcium/1,000 kcal, tertiles** |  |  |  |  |  |  |  |  |  |  |  |  |  |
| 1 | 34 | 4,582 | (3,999, 5,165) | - |  | 2,361 | (1,228, 3,494) | - |  | 2,723 | (2,361, 3,086) | - |  |
| 2 | 35 | 4,259 | (3,664, 4,854) | -7.0 |  | 2,363 | (1,207, 3,519) | 0.1 |  | 2,526 | (2,156, 2,896) | -7.2 |  |
| 3 | 35 | 4,911 | (4,316, 5,506) | 7.2 | 0.36 | 2,251 | (1,094, 3,408) | -4.7 | 0.99 | 2,891 | (2,521, 3,261) | 6.2 | 0.44 |
| **Dietary fiber/1,000 kcal, tertiles** |  |  |  |  |  |  |  |  |  |  |  |  |  |
| 1 | 34 | 4,517 | (3,809, 5,225) | - |  | 2,072 | (783, 3,360) | - |  | 2,741 | (2,301, 3,181) | - |  |
| 2 | 35 | 4,449 | (3,879, 5,019) | -1.5 |  | 1,143 | (107, 2,180) | -44.8 |  | 2,648 | (2,294, 3,002) | -3.4 |  |
| 3 | 35 | 4,793 | (4,064, 5,522) | 6.1 | 0.79 | 3,752 | (2,424, 5,079) | 81.1 | 0.02 | 2,759 | (2,305, 3,212) | 0.7 | 0.91 |
| **Total meat intake, tertiles** |  |  |  |  |  |  |  |  |  |  |  |  |  |
| 1 | 34 | 4,810 | (4,106, 5,514) | - |  | 1,546 | (217, 2,874) | - |  | 2,798 | (2,359, 3,238) | - |  |
| 2 | 35 | 4,336 | (3,778, 4,895) | -9.9 |  | 3,113 | (2,059, 4,167) | 101.4 |  | 2,633 | (2,284, 2,981) | -5.9 |  |
| 3 | 35 | 4,636 | (3,976, 5,296) | -3.6 | 0.53 | 2,234 | (988, 3,479) | 44.5 | 0.16 | 2,724 | (2,312, 3,136) | -2.6 | 0.82 |
| **Total vegetables and fruit intake, tertiles** |  |  |  |  |  |  |  |  |  |  |  |  |  |
| 1 | 34 | 4,130 | (3,536, 4,723) | - |  | 3,022 | (1,900, 4,143) | - |  | 2,427 | (2,057, 2,798) | - |  |
| 2 | 35 | 4,820 | (4,267, 5,372) | 16.7 |  | 1,417 | (373, 2,461) | -53.1 |  | 2,810 | (2,465, 3,154) | 15.8 |  |
| 3 | 35 | 4,798 | (4,213, 5,384) | 16.2 | 0.22 | 2,589 | (1,482, 3,696) | -14.3 | 0.12 | 2,904 | (2,538, 3,270) | 19.7 | 0.20 |
| **Serum 25-OH-vitamin D (ng/mL)** |  |  |  |  |  |  |  |  |  |  |  |  |  |
| < 17.9 | 34 | 4,752 | (4,185, 5,324) | - |  | 2,787 | (1,713, 3,862) | - |  | 2,837 | (2,485, 3,189) | - |  |
| 17.9 – 26.9 | 35 | 4,293 | (3,718, 4,867) | -9.7 |  | 1,413 | (328, 2,497) | -49.3 |  | 2,511 | (2,156, 2,867) | -11.5 |  |
| > 26.9 | 35 | 4,719 | (4,170, 5,268) | -0.7 | 0.50 | 2,787 | (1,751, 3,824) | 0.0 | 0.16 | 2,801 | (2,462, 3,141) | -1.3 | 0.42 |

Abbreviations: CI, confidence interval; NSAID, non-steroidal anti-inflammatory drug; OD, optical density; Prop. diff., proportional difference.

^a^ Biomarker expression measured using automated immunohistochemistry with image analysis. Adjusted mean differences in optical densities of biomarker labeling were calculated using general linear models, adjusted for staining batch, total energy intake, and total vegetables and fruit intake).

^b^ Measured using automated immunohistochemistry with image analysis.

^c^ Calculated as (comparison group mean - reference group mean) / (reference group mean) x 100%.

^d^ Take at least once a week.

^e^ Low alcohol consumption is ≤ 0.8 drinks/day for men, 0 drinks/day for women; high alcohol consumption is > 0.8 drinks/day for men, > 0 drinks/day for women.

^f^ Dietary plus supplemental intake.

**Supplemental Table 5.** Comparisons^a^ of adjusted mean MSH2 expression^b^ in the normal-appearing colorectal mucosa of sporadic colorectal adenoma patients (*n* = 104), by selected participant characteristics.

| **Characteristics** | ***n*** | | **Whole crypts, mean (OD)** | | **95% CI** | | **Prop. diff.**^c^ **(%)** | | ***p*** | | **Upper 40% of crypts, mean (OD)** | | **95% CI** | | **Prop. diff.**^c^ **(%)** | | ***p*** | | **Lower 60% of crypts, mean (OD)** | | **95% CI** | | **Prop. diff.**^c^ **(%)** | | ***p*** | |  |
| --- | --- | --- | --- | --- | --- | --- | --- | --- | --- | --- | --- | --- | --- | --- | --- | --- | --- | --- | --- | --- | --- | --- | --- | --- | --- | --- | --- |
| **Age (yrs)** |  | |  | |  | |  | |  | |  | |  | |  | |  | |  | |  | |  | |  | |  |
| 47 – 54 | 34 | | 1,862 | | (1,616, 2,108) | | - | |  | | 357 | | (-528, 1,242) | | - | |  | | 1,468 | | (1,279, 1,656) | | - | |  | |  |
| 55 – 62 | 35 | | 1,878 | | (1,643, 2,114) | | 0.9 | |  | | 245 | | (-603, 1,093) | | -31.4 | |  | | 1,498 | | (1,318, 1,679) | | 2.0 | |  | |  |
| 63 – 75 | 35 | | 1,887 | | (1,644, 2,130) | | 1.3 | | 0.99 | | 947 | | (72, 1,821) | | 165.3 | | 0.51 | | 1,463 | | (1,277, 1,650) | | -0.3 | | 0.96 | |  |
| **Sex** |  | |  | |  | |  | |  | |  | |  | |  | |  | |  | |  | |  | |  | |  |
| Male | 48 | | 1,944 | | (1,550, 2,338) | | - | |  | | 495 | | (-938, 1,928) | | - | |  | | 1,562 | | (1,261, 1,864) | | - | |  | |  |
| Female | 56 | | 1,819 | | (1,483, 2,155) | | -6.4 | | 0.72 | | 545 | | (-677, 1,767) | | 10.1 | | 0.97 | | 1,404 | | (1,146, 1,661) | | -10.1 | | 0.55 | |  |
| **Regularly**^d^ **take aspirin** |  | |  | |  | |  | |  | |  | |  | |  | |  | |  | |  | |  | |  | |  |
| No | 64 | | 2,020 | | (1,816, 2,224) | | - | |  | | 430 | | (-312, 1,171) | | - | |  | | 1,575 | | (1,419, 1,732) | | - | |  | |  |
| Yes | 40 | | 1,732 | | (1,575, 1,888) | | -14.3 | | 0.04 | | 615 | | (46, 1,184) | | 43.0 | | 0.71 | | 1,376 | | (1,256, 1,497) | | -12.6 | | 0.06 | |  |
| **Regularly**^d^ **take other NSAID** |  | |  | |  | |  | |  | |  | |  | |  | |  | |  | |  | |  | |  | |  |
| No | 69 | | 1,946 | | (1,726, 2,167) | | - | |  | | 910 | | (114, 1,707) | | - | |  | | 1,528 | | (1,359, 1,697) | | - | |  | |  |
| Yes | 35 | | 1,841 | | (1,689, 1,992) | | -5.4 | | 0.45 | | 331 | | (-216, 878) | | -63.6 | | 0.25 | | 1,450 | | (1,334, 1,566) | | -5.1 | | 0.46 | |  |
| **Currently smoke** |  | |  | |  | |  | |  | |  | |  | |  | |  | |  | |  | |  | |  | |  |
| No | 96 | | 1,874 | | (1,748, 2,000) | | - | |  | | 588 | | (135, 1,041) | | - | |  | | 1,477 | | (1,381, 1,574) | | - | |  | |  |
| Yes | 8 | | 1,896 | | (1,418, 2,375) | | 1.2 | | 0.93 | | -433 | | (-2,157, 1,291) | | -173.6 | | 0.26 | | 1,457 | | (1,090, 1,824) | | -1.4 | | 0.92 | |  |
| **Alcohol consumption** |  | |  | |  | |  | |  | |  | |  | |  | |  | |  | |  | |  | |  | |  |
| Low^e^ | 57 | | 1,914 | | (1,731, 2,097) | |  | |  | | 527 | | (-140, 1,195) | |  | |  | | 1,501 | | (1,361, 1,641) | |  | |  | |  |
| High^e^ | 47 | | 1,756 | | (1,549, 1,962) | | -8.3 | | 0.31 | | 563 | | (-192, 1,318) | | 6.8 | | 0.95 | | 1,395 | | (1,236, 1,553) | | -7.1 | | 0.38 | |  |
| **Physical activity** |  | |  | |  | |  | |  | |  | |  | |  | |  | |  | |  | |  | |  | |  |
| Low | 30 | | 1,815 | | (1,569, 2,062) | | - | |  | | 499 | | (-392, 1,389) | | - | |  | | 1,429 | | (1,240, 1,617) | | - | |  | |  |
| Moderate | 38 | | 1,647 | | (1,723, 2,171) | | -9.3 | |  | | 159 | | (-651, 968) | | -68.1 | |  | | 1,530 | | (1,358, 1,701) | | 7.1 | |  | |  |
| High | 27 | | 1,871 | | (1,666, 2,076) | | 3.1 | | 0.74 | | 850 | | (109, 1,592) | | 70.3 | | 0.46 | | 1,474 | | (1,317, 1,631) | | 3.1 | | 0.74 | |  |
| **Body mass index (kg/m^2^)** |  | |  | |  | |  | |  | |  | |  | |  | |  | |  | |  | |  | |  | |  |
| < 25.0 | 22 | | 1,859 | | (1,572, 2,147) | | - | |  | | 1,419 | | (399, 2,439) | | - | |  | | 1,486 | | (1,266, 1,706) | | - | |  | |  |
| 25.0 – 29.9 | 43 | | 1,921 | | (1,721, 2,121) | | 3.3 | |  | | 381 | | (-329, 1,091) | | -73.2 | |  | | 1,508 | | (1,354, 1,661) | | 1.5 | |  | |  |
| ≥ 30 | 39 | | 1,836 | | (1,625, 2,047) | | -1.2 | | 0.84 | | 178 | | (-572, 928) | | -87.5 | | 0.15 | | 1,437 | | (1,275, 1,599) | | -3.3 | | 0.83 | |  |
| **Total energy, tertiles** | |  | |  | |  | |  | |  | |  | |  | |  | |  | |  | |  | |  | |  | |
| 1 | 34 | | 1,823 | | (1,591, 2,055) | | - | |  | | 920 | | (86, 1,754) | | - | |  | | 1,413 | | (1,235, 1,591) | | - | |  | |  |
| 2 | 35 | | 1,873 | | (1,646, 2,100) | | 2.7 | |  | | 63 | | (-752, 878) | | -93.2 | |  | | 1,484 | | (1,310, 1,658) | | 5.0 | |  | |  |
| 3 | 35 | | 1,928 | | (1,701, 2,156) | | 5.8 | | 0.82 | | 605 | | (-212, 1,421) | | -34.2 | | 0.36 | | 1,527 | | (1,353, 1,701) | | 8.1 | | 0.06 | |  |
| **Saturated fat (% of total energy), tertiles** |  | |  | |  | |  | |  | |  | |  | |  | |  | |  | |  | |  | |  | |  |
| 1 | 34 | | 1,930 | | (1,700, 2,160) | | - | |  | | 305 | | (-530, 1,140) | | - | |  | | 1,527 | | (1,351, 1,703) | | - | |  | |  |
| 2 | 35 | | 1,876 | | (1,652, 2,101) | | -2.8 | |  | | 676 | | (-140, 1,493) | | 121.6 | |  | | 1,492 | | (1,320, 1,664) | | -2.3 | |  | |  |
| 3 | 35 | | 1,819 | | (1,569, 2,069) | | -5.8 | | 0.83 | | 583 | | (-325, 1,491) | | 91.1 | | 0.81 | | 1,408 | | (1,216, 1,599) | | -7.8 | | 0.69 | |  |
| **Total fat/1,000 kcal, tertiles** |  | |  | |  | |  | |  | |  | |  | |  | |  | |  | |  | |  | |  | |  |
| 1 | 34 | | 1,892 | | (1,663, 2,120) | | - | |  | | 297 | | (-515, 1,109) | | - | |  | | 1,497 | | (1,322, 1,672) | | - | |  | |  |
| 2 | 35 | | 1,870 | | (1,643, 2,097) | | -1.2 | |  | | 1,122 | | (315, 1,929) | | 277.8 | |  | | 1,471 | | (1,297, 1,645) | | -1.7 | |  | |  |
| 3 | 35 | | 1,865 | | (1,634, 2,097) | | -1.4 | | 0.99 | | 135 | | (-688, 957) | | -54.5 | | 0.22 | | 1,460 | | (1,283, 1,637) | | -2.5 | | 0.96 | |  |
| **Total**^f^ **vitamin E/1,000 kcal, tertiles** |  | |  | |  | |  | |  | |  | |  | |  | |  | |  | |  | |  | |  | |  |
| 1 | 34 | | 1,933 | | (1,690, 2,176) | | - | |  | | 289 | | (-594, 1,172) | | - | |  | | 1,527 | | (1,340, 1,713) | | - | |  | |  |
| 2 | 35 | | 1,803 | | (1,575, 2,031) | | -6.7 | |  | | 450 | | (-378, 1,278) | | 55.7 | |  | | 1,427 | | (1,252, 1,602) | | -6.5 | |  | |  |
| 3 | 35 | | 1,892 | | (1,657, 2,128) | | -2.1 | | 0.73 | | 809 | | (-46, 1,665) | | 179.9 | | 0.72 | | 1,476 | | (1,295, 1,656) | | -3.3 | | 0.75 | |  |
| **Total**^f^ **calcium/1,000 kcal, tertiles** |  | |  | |  | |  | |  | |  | |  | |  | |  | |  | |  | |  | |  | |  |
| 1 | 34 | | 2,073 | | (1,840, 2,306) | | - | |  | | 406 | | (-447, 1,260) | | - | |  | | 1,595 | | (1,416, 1,774) | | - | |  | |  |
| 2 | 35 | | 1,722 | | (1,462, 1,982) | | -16.9 | |  | | 752 | | (-201, 1,705) | | 85.2 | |  | | 1,369 | | (1,169, 1,569) | | -14.2 | |  | |  |
| 3 | 35 | | 1,914 | | (1,574, 2,055) | | -7.7 | | 0.14 | | 433 | | (-448, 1,315) | | 6.7 | | 0.86 | | 1,452 | | (1,267, 1,637) | | -9.0 | | 0.11 | |  |
| **Dietary fiber/1,000 kcal, tertiles** |  | |  | |  | |  | |  | |  | |  | |  | |  | |  | |  | |  | |  | |  |
| 1 | 34 | | 1,932 | | (1,671, 2,192) | | - | |  | | 480 | | (-456, 1,416) | | - | |  | | 1,506 | | (1,306, 1,706) | | - | |  | |  |
| 2 | 35 | | 1,732 | | (1,507, 1,957) | | -10.4 | |  | | 1,145 | | (334, 1,955) | | 138.5 | |  | | 1,381 | | (1,208, 1,555) | | -8.3 | |  | |  |
| 3 | 35 | | 1,965 | | (1,705, 2,225) | | 1.7 | | 0.32 | | -72 | | (-1,008, 863) | | -115.0 | | 0.15 | | 1,542 | | (1,342, 1,742) | | 2.4 | | 0.43 | |  |
| **Total meat intake, tertiles** |  | |  | |  | |  | |  | |  | |  | |  | |  | |  | |  | |  | |  | |  |
| 1 | 34 | | 1,957 | | (1,675, 2,239) | | - | |  | | 1,282 | | (277, 2,286) | | - | |  | | 1,528 | | (1,311, 1,744) | | - | |  | |  |
| 2 | 35 | | 1,964 | | (1,733, 2,195) | | 0.4 | |  | | -105 | | (-928, 718) | | -108.2 | |  | | 1,556 | | (1,379, 1,733) | | 1.8 | |  | |  |
| 3 | 35 | | 1,731 | | (1,467, 1,996) | | -11.5 | | 0.45 | | 457 | | (-484, 1,398) | | -64.4 | | 0.10 | | 1,363 | | (1,160, 1,565) | | -10.8 | | 0.41 | |  |
| **Total vegetables and fruit intake, tertiles** |  | |  | |  | |  | |  | |  | |  | |  | |  | |  | |  | |  | |  | |  |
| 1 | 34 | | 1,882 | | (1,632, 2,132) | | - | |  | | 205 | | (-683, 1,094) | | - | |  | | 1,482 | | (1,290, 1,674) | | - | |  | |  |
| 2 | 35 | | 1,946 | | (1,724, 2,167) | | 3.4 | |  | | 1,193 | | (406, 1,981) | | 482.0 | |  | | 1,522 | | (1,352, 1,692) | | 2.7 | |  | |  |
| 3 | 35 | | 1,791 | | (1,550, 2,031) | | -4.8 | | 0.66 | | 73 | | (-782, 929) | | -64.4 | | 0.13 | | 1,418 | | (1,233, 1,603) | | -4.3 | | 0.73 | |  |
| **Serum 25-OH-vitamin D (ng/mL)** |  | |  | |  | |  | |  | |  | |  | |  | |  | |  | |  | |  | |  | |  |
| < 17.9 | 34 | | 1,863 | | (1,631, 2,096) | | - | |  | | 114 | | (-726, 954) | | - | |  | | 1,465 | | (1,286, 1,644) | | - | |  | |  |
| 17.9 – 26.9 | 35 | | 1,784 | | (1,547, 2,021) | | -4.2 | |  | | 1,007 | | (150, 1,864) | | 783.3 | |  | | 1,418 | | (1,235, 1,600) | | -3.2 | |  | |  |
| > 26.9 | 35 | | 1,984 | | (1,742, 2,225) | | 6.5 | | 0.54 | | 444 | | (-429, 1,316) | | 289.5 | | 0.37 | | 1,548 | | (1,362, 1,733) | | 5.7 | | 0.64 | |  |

Abbreviations: CI, confidence interval; NSAID, non-steroidal anti-inflammatory drug; OD, optical density; Prop. diff., proportional difference.

^a^ Biomarker expression measured using automated immunohistochemistry with image analysis. Adjusted mean differences in optical densities of biomarker labeling were calculated using general linear models, adjusted for staining batch, total energy intake, aspirin use, and total calcium intake/1,000 kcal of total energy intake.

^b^ Measured using automated immunohistochemistry with image analysis.

^c^ Calculated as (comparison group mean - reference group mean) / (reference group mean) x 100%.

^d^ Take at least once a week.

^e^ Low alcohol consumption is ≤ 0.8 drinks/day for men, 0 drinks/day for women; high alcohol consumption is > 0.8 drinks/day for men, > 0 drinks/day for women.

^f^ Dietary plus supplemental intake.
